# Genome-wide identification and characterization of the 14–3-3 family in *Vitis vinifera* L. during berry development and cold- and heat-stress response

**DOI:** 10.1186/s12864-018-4955-8

**Published:** 2018-08-02

**Authors:** Cheng Cheng, Yi Wang, Fengmei Chai, Shaohua Li, Haiping Xin, Zhenchang Liang

**Affiliations:** 1 Key Laboratory of Plant Germplasm Enhancement and Specialty Agriculture, Wuhan Botanical Garden, The Chinese Academy of Sciences, Wuhan, People’s Republic of China; 2 Beijing Key Laboratory of Grape Sciences and Enology, Laboratory of Plant Resources, Institute of Botany, the Chinese Academy of Sciences, Beijing, People’s Republic of China; 3 University of Chinese Academy of Sciences, Beijing, People’s Republic of China; 4 Sino-Africa Joint Research Center, Chinese Academy of Sciences, Wuhan, People’s Republic of China

**Keywords:** Abiotic stress, Berry, Development, Expression, Grape, 14–3-3

## Abstract

**Background:**

The 14–3-3 family of ubiquitous proteins in eukaryotes plays important roles in the regulation of various plant biological processes. However, less information is known about this family in grape fruit.

**Results:**

To investigate the characteristics and functions of 14–3-3 in grape, a total of 11 14–3-3 proteins were identified. Phylogenetic analysis of 14–3-3 proteins in grape (VviGRFs) with homologous proteins in *Arabidopsis* showed that these proteins were classified into two groups, namely, epsilon and non-epsilon groups. Epsilon group members commonly contained more introns and motifs than non-epsilon group, and some intron positions were found to be conserved between *Vitis* and *Arabidopsis* 14–3-3 genes. RNA-seq and qRT-PCR results indicated that *VviGRF* genes may be involved in the regulation of grape development and berry ripening. Moreover, six *VviGRF*s exhibited significantly up- or down-regulated expression in response to cold and heat stresses, thereby revealing their potential roles in the regulation of abiotic stress responses.

**Conclusions:**

This work provides fundamental knowledge for further studies about the biological roles of *VviGRF*s in grape development and abiotic stress response. The present result will also be beneficial for understanding their molecular mechanisms and improving grape agricultural traits in the future.

**Electronic supplementary material:**

The online version of this article (10.1186/s12864-018-4955-8) contains supplementary material, which is available to authorized users.

## Background

The 14–3-3 proteins were first found in bovine brain and named according to their elution and migration pattern on 2D DEAE-cellulose chromatography and starch gel electrophoresis [1]. This proteins, as highly conserved regulatory proteins, are ubiquitous in eukaryotes, including plants, insects, mammals, amphibians and yeasts [2–5]. Generally, these proteins exist as homo- or hetero-isoform dimers, which form dimer groove structures [6]. The structures provide binding sites for 14–3-3 proteins to interact with their targets, such as enzymes involved in primary biosynthetic and energy metabolism (e.g. nitrate reductase, brassinosteroid receptor kinase and H^+^-ATPase) and signal proteins (e.g. lipoxygenase and protein kinases) [7–9]. With the interaction, 14–3-3 proteins function as scaffolding proteins that are involved in the regulation of diverse biological processes, including carbon and nitrogen metabolisms, tricarboxylic acid cycle and the shikimate pathway [8, 10].

To date, 8, 12, 25, 25, 13, 18 and 10 14–3-3 proteins were identified in rice, tomato, cotton, banana, *Arabidopsis*, soybean and *Hevea brasiliensis*, respectively [11–17]. Previous studies revealed that 14–3-3 proteins play important roles in plant growth and development. For example, most banana *MaGRF*s show an accumulated transcription during fruit development and post-harvest ripening [14]. Various *GhGRF* interaction partners identified in cotton fibre are involved in plant development, metabolism, signalling transduction and other cellular processes. The overexpression of *GhGRFl* promotes cotton fibre elongation and maturation [18]. In *Arabidopsis*, antisense technology revealed that *AtGRF10* and *AtGRF9* regulate starch accumulation [19]. The 14–3-3 proteins are also related to various abiotic stress responses. In rice, most *OsGRF* expression changes in response to heat, low temperature and salt stresses [11]. Overexpressed *AtGRF6* transgenic cotton shows a stay-green phenotype and improved tolerance to drought stress [20].

Grape (*Vitis vinifera* L.) is one of the most important fruit crops worldwide; this crop has been widely cultivated because of its nutritional value and wide application [21]. The grape production is often threatened with abiotic stresses, such as heat or low temperature, drought and salt stresses. Currently, the14–3-3 family in grape has not been studied systematically, except for two 14–3-3 genes (*Vv1CS* and *Vv2CS*) that are cloned from *V. vinifera* cv. ‘Cabernet Sauvignon’; nevertheless, the functions of these genes are still unclear [22]. Several unknown grape 14–3-3-like proteins are also involved in biotic and abiotic stress responses [23]. Thus, 14–3-3 gene family should be systematically analysed to characterise the expression during development and response to abiotic stress in grape.

In the present study, the identification, phylogeny, structure and gene location and duplication were performed on the 14–3-3 family in grape. The expression levels of *14–3-3* s in various tissues and organs of grape during different developmental stages and responses to heat and cold stresses were systematically studied in detail. The present results will provide important information for further study of the regulation mechanism of *14–3-3* s in grape berry development and under abiotic stress.

## Methods

### Identification of 14–3-3 proteins in grape

The 14–3-3 proteins in grape were identified according to 12X V1 version (Grape Genome Database, http://genomes.cribi.unipd.it/DATA/, Table 1). The 14–3-3 protein sequences of *Oryza. sativa* and *A. thaliana* used in this study were obtained from NCBI (http://www.ncbi.nlm.nih.gov/).

**Table 1:** The identification of 14–3-3 members in grape

Group	Gene	Locus ID	Chromosome Location	Strand	No. of exons	protein		
						Length (aa)	PI	Mol wt (kDa)
Epsilon group	*VviGRF9a*	VIT_10s0003g01240	chr10: 2568369–2,571,848	forward	6	287	4.8	32.38
	*VviGRF9b*	VIT_19s0014g01420	chr19: 1489168–1,494,033	forward	7	265	4.75	29.93
	*VviGRF11*	VIT_18s0001g05720	chr18: 4453327–4,457,820	reverse	7	253	4.79	28.74
	*VviGRF12*	VIT_01s0011g00620	chr1: 563253–566,683	reverse	7	266	4.84	30.16
Non-epsilon group	*VviGRF14*	VIT_18s0001g07240	chr18: 5477686–5,481,158	reverse	4	253	4.8	28.65
	*VviGRF15*	VIT_00s0199g00190	chrUn: 11210066–11,213,944	forward	4	254	4.8	28.81
	*VviGRF16*	VIT_14s0006g03230	chr14: 21820857–21,823,454	forward	4	263	4.74	29.54
	*VviGRF17*	VIT_18s0001g06330	chr18: 4742712–4,744,730	forward	4	261	4.68	29.34
	*VviGRF18*	VIT_07s0191g00090	chr7: 14850330–14,852,315	forward	4	256	4.76	28.78
	*VviGRF-like1*	VIT_00s0199g00140	chrUn: 11185021–11,188,247	forward	2	582	5.95	66.03
	*VviGRF-like2*	VIT_02s0033g00780	chr2: 15461190–15,461,553	reverse	1	64	7.57	6.91

ChrUn is the unanchored chromosome. Locus ID listed from 12X V1 version. Information of VviGRF proteins was searched by Swissprot website. Strand means the direction of transcription. More detail information of grape 14–3-3 family can be found in Additional file 10

Hidden Markov Model (HMM), BLASTP program and NCBI-Conserved Domain Data (CDD) search were used to identify the 14–3-3 members in grape. HMM was constructed to scan 14–3-3 proteins in grape by using the obtained OsGRF and AtGRF protein sequences. OsGRFs, AtGRFs, together with 14–3-3 proteins published in NCBI were used as query sequences to blast against the grape protein database obtained from the 12X V1 version. Finally, 11 proteins containing 14–3-3 domain were identified as members of 14–3-3 in grape using NCBI-CDD (http://www.ncbi.nlm.nih.gov/Structure/cdd/wrpsb.cgi) [24].

### Multiple sequence alignment and phylogenetic analysis

**Fig. 1:**
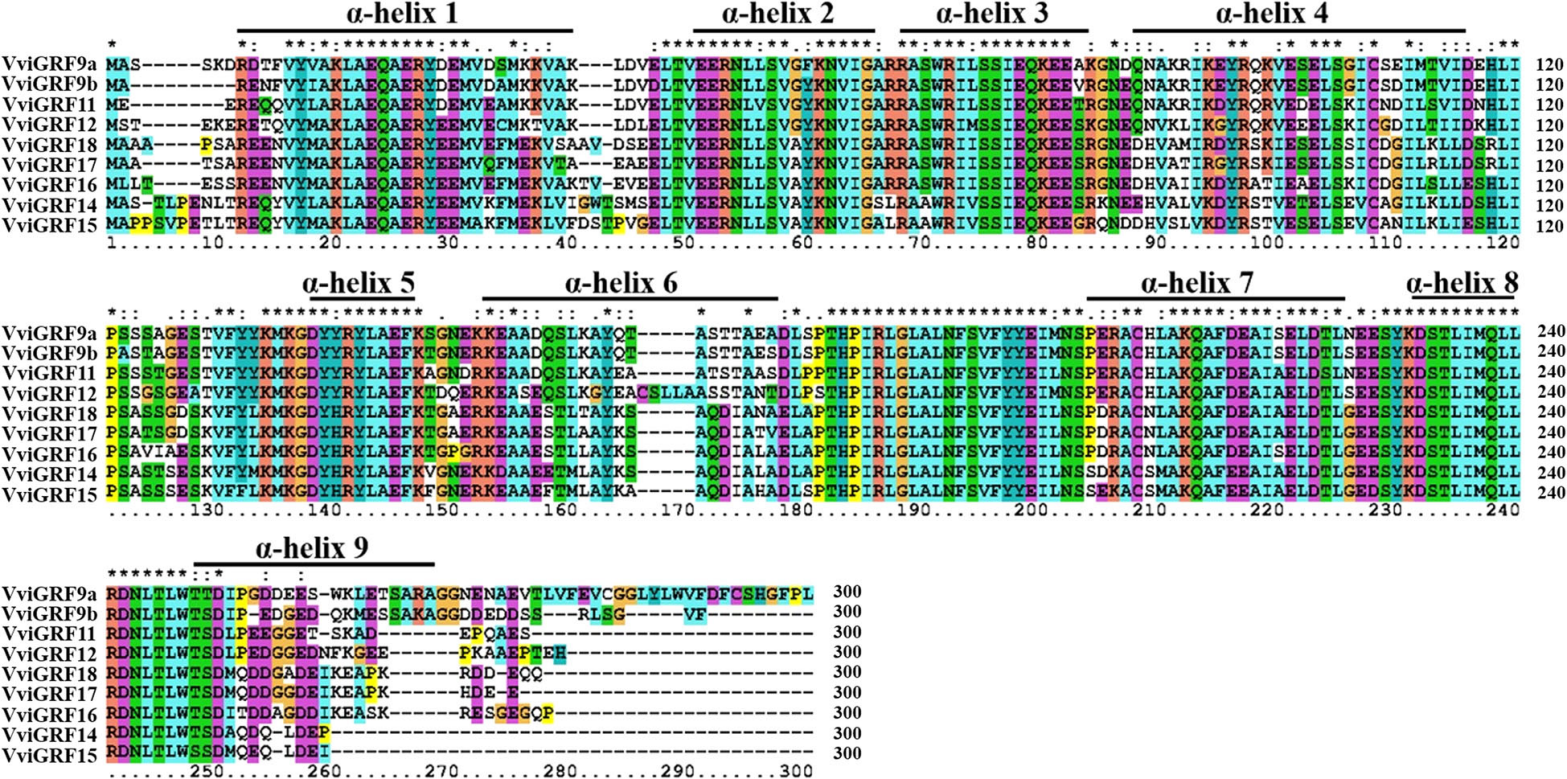
Multiple sequence alignment of VviGRFs. VviGRF proteins were aligned using ClustalX2 program. ɑ helices of VviGRF proteins were analysed by searching SOPMA website, and were marked on their corresponding sequences. VviGRF-like1 and VviGRF-like2 respectively are the longest and the shortest amino acid sequences among VviGRFs, and they show very different motif and intron-exon structures with other 14–3-3 members (Fig. 3b). In order to present a more visualized secondary structure of VviGRFs, nine VviGRF proteins were used except for VviGRF-like1 and VviGRF-like2

**Fig. 2:**
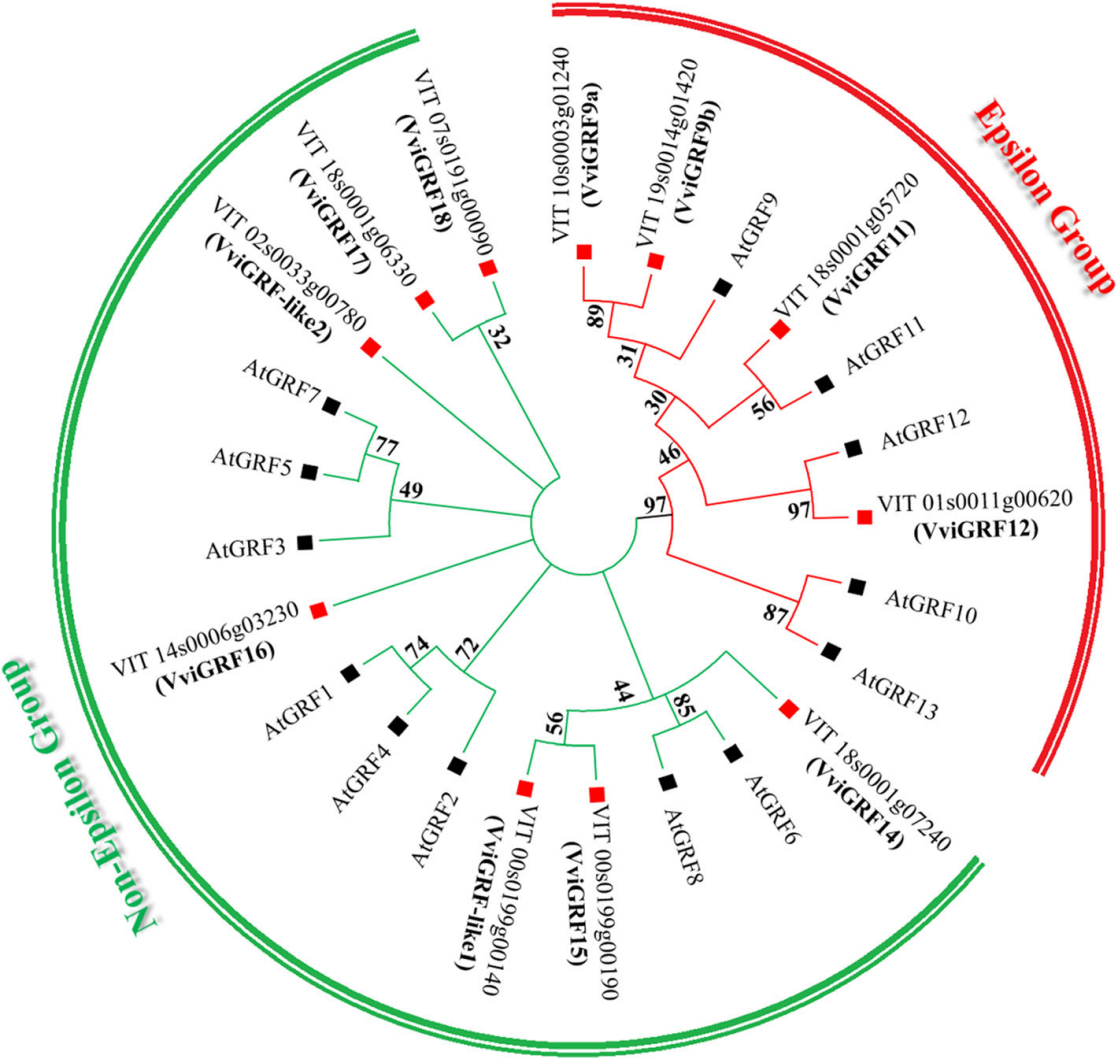
Phylogenetic analysis of 14–3-3 members in *V. vinifera* and *Arabidopsis.* Phylogenetic tree was constructed by MEGA7 with Neighbour-Joining method and bootstrap of 1000 replications. Grape 14–3-3 s were named by reference to their domains and the grapevine gene nomenclature system [28]. Values less than 40 were cut off

**Fig. 3:**
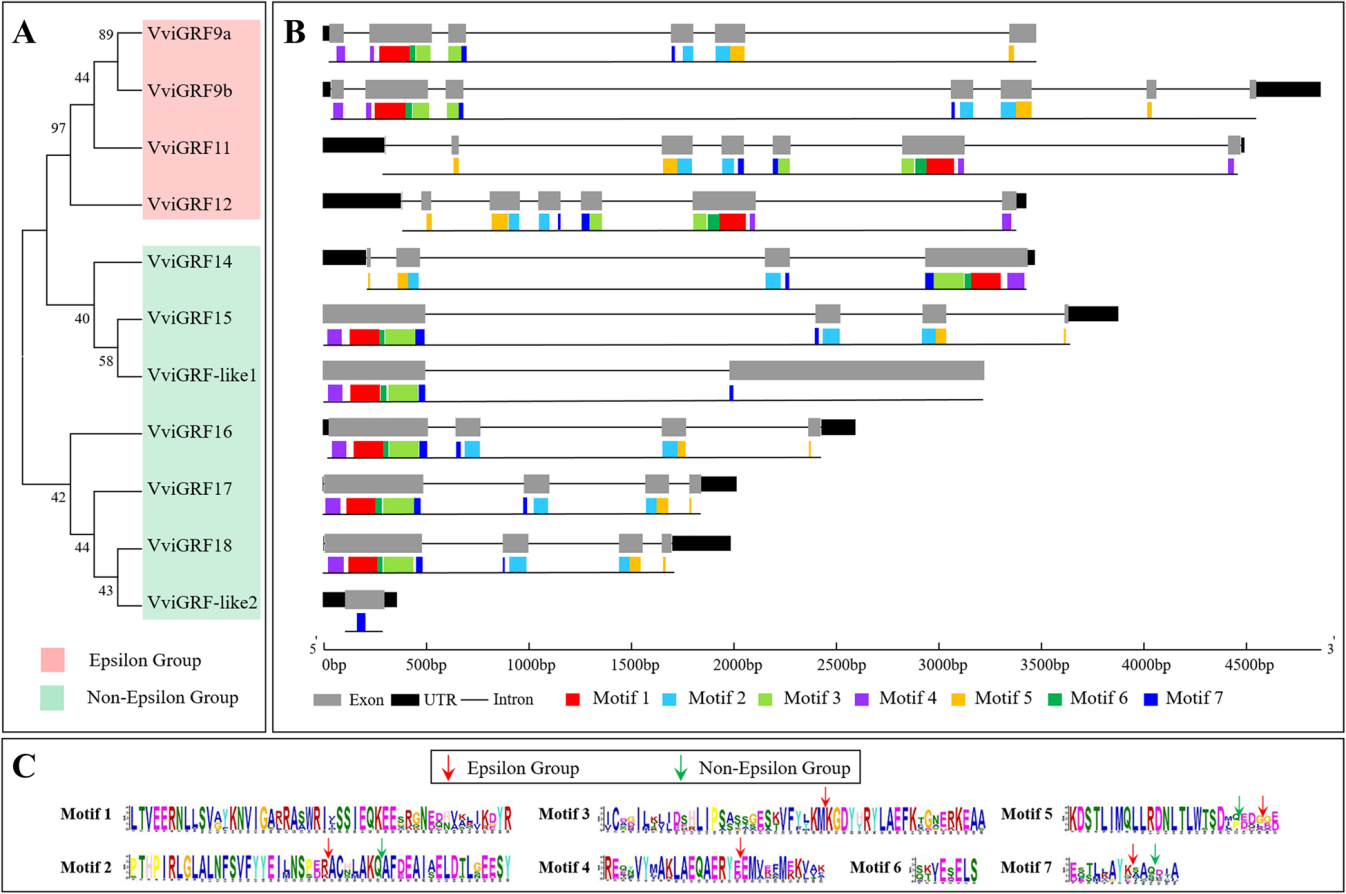
Phylogenetic relationship, motif and gene structure analysis of grape 14–3-3 members. (**a**) Phylogenetic tree of 14–3-3 family in *V. vinifera*. Bootstrap values less than 40 were cut off. (**b**) Gene structure and motif analysis of grape 14–3-3 members. The black blocks represent the untranslated region (UTR), the gray blocks represent exons, and the black lines represent introns. Different motifs were represent in different colour blocks, and their sequences were list in **c**. (**c**) The amino acid sequences of motifs in VviGRF proteins. Arrows showed the intron position appeared in “exon-intron-exon” sequences. Red arrow means the epsilon group, green arrow means the non-epsilon group. The detail information for the intron position was shown in Additional file 7

Identified 14–3-3 proteins in grape were aligned with AtGRFs using ClustalX2 program [25]. The secondary structures of VviGRF proteins were analysed by searching in Self-Optimized Prediction Methods with Alignment (SOPMA, https://npsa-prabi.ibcp.fr/cgi-bin/npsa_automat.pl?page=npsa_sopma.html) [26]. With the combined aligned results, typical α helices in 14–3-3 proteins were marked on their corresponding sequences (Fig. 1). Phylogenetic analysis was conducted by MEGA7 based on the aligned results [27]. Neighbour-joining method was used with bootstrap replications of 1000 (Figs. 2 and 3a). 11 VviGRF proteins were named based on their domains and their phylogenetic relationships with AtGRFs [28]. When a one-to-one orthology was present in the *Arabidopsis* 14–3-3 family, the grape 14–3-3 members were given the corresponding *Arabidopsis*-like name (e.g., AtGRF11 and VviGRF11). Two grape genes had the same phylogenetic distance from a single homologue in *Arabidopsis* were differentiated by a number (e.g., AtGRF9, VviGRF9a and VviGRF9b). As for remaining 14–3-3 members, there are no *Arabidopsis* genes homologous to them, thus we named them on the basis of domain analysis: both VIT_00s0199g00140 and VIT_02s0033g00780 have incomplete 14–3-3 domains, thus these two protein were named as VviGRF-like1 and –like2. Since there are only 13 AtGRFs in *Arabidopsis* 14–3-3 family, other five members in several adjacent subclasses share the same 14–3-3 domain were named from VviGRF14 to 15, 16, 17 and 18. The isoelectric point (PI) and molecular weight (Mol wt) of VviGRF proteins were examined on Swiss-Prot website (http://web.expasy.org/compute_pi/, Table 1).

### Gene structure and motif analysis

The gene structure data used in this study were obtained from the 12X V1 genome annotation file (http://genomes.cribi.unipd.it/DATA/) and The Arabidopsis Information Resource website (TAIR, http://www.arabidopsis.org/browse/genefamily/14-3-3.jsp). Gene structure analysis was conducted using Gene Structure Display Server version 2.0 (http://gsds.cbi.pku.edu.cn/, Fig. 3b) [29]. According to the amino acid sequences, the motifs of VviGRF proteins were analysed by using MEME program (http://meme-suite.org/tools/meme) [30]. The motif distribution type was zero or one occurrence per sequence, and only motifs with E-value > 0.05 were present (Fig. 3b and Additional file 1).

### Gene location and duplication

**Fig. 4:**
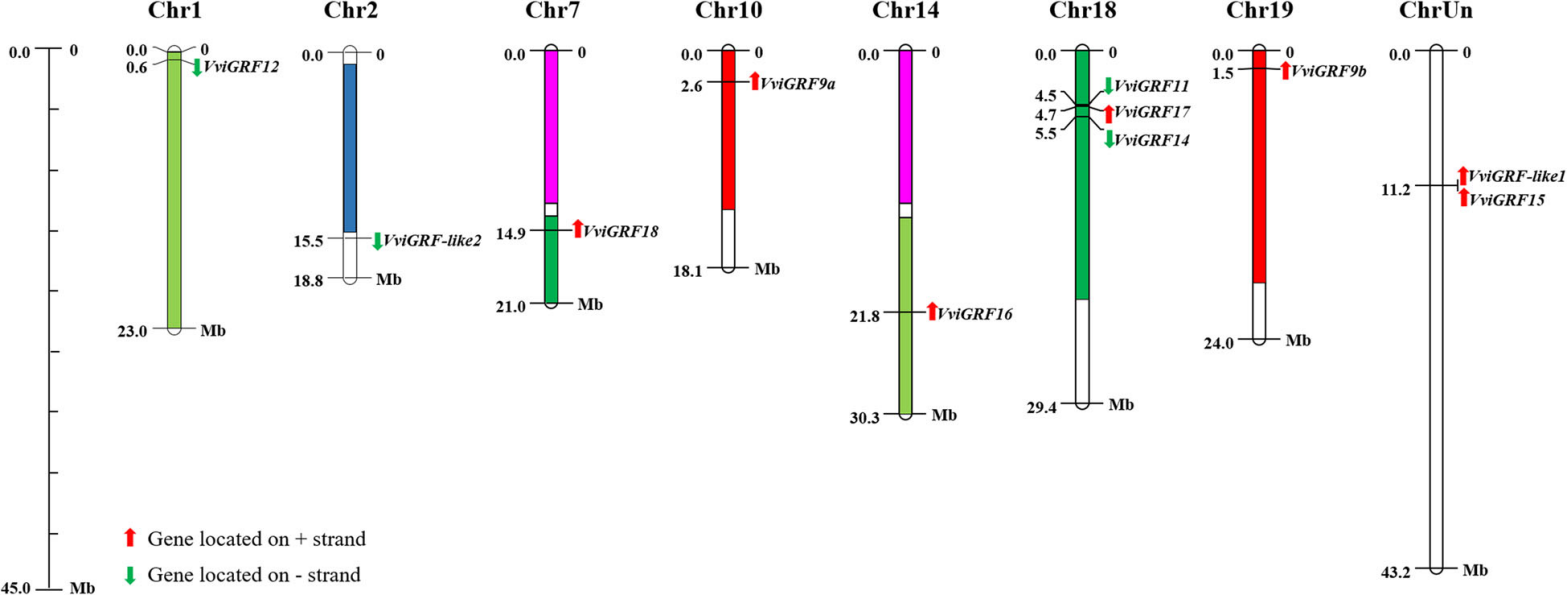
The chromosome distribution of *VviGRF* genes. Only chromosomes contained *VviGRF* genes are represented in this figure. The chromosome numbers and sizes (Mb) are indicated at the top and bottom of each chromosome, respectively. Coloured regions in chromosomes indicate whole genomic duplication [52]. The red and green arrows next to genes show the direction of their transcription

**Fig. 5:**
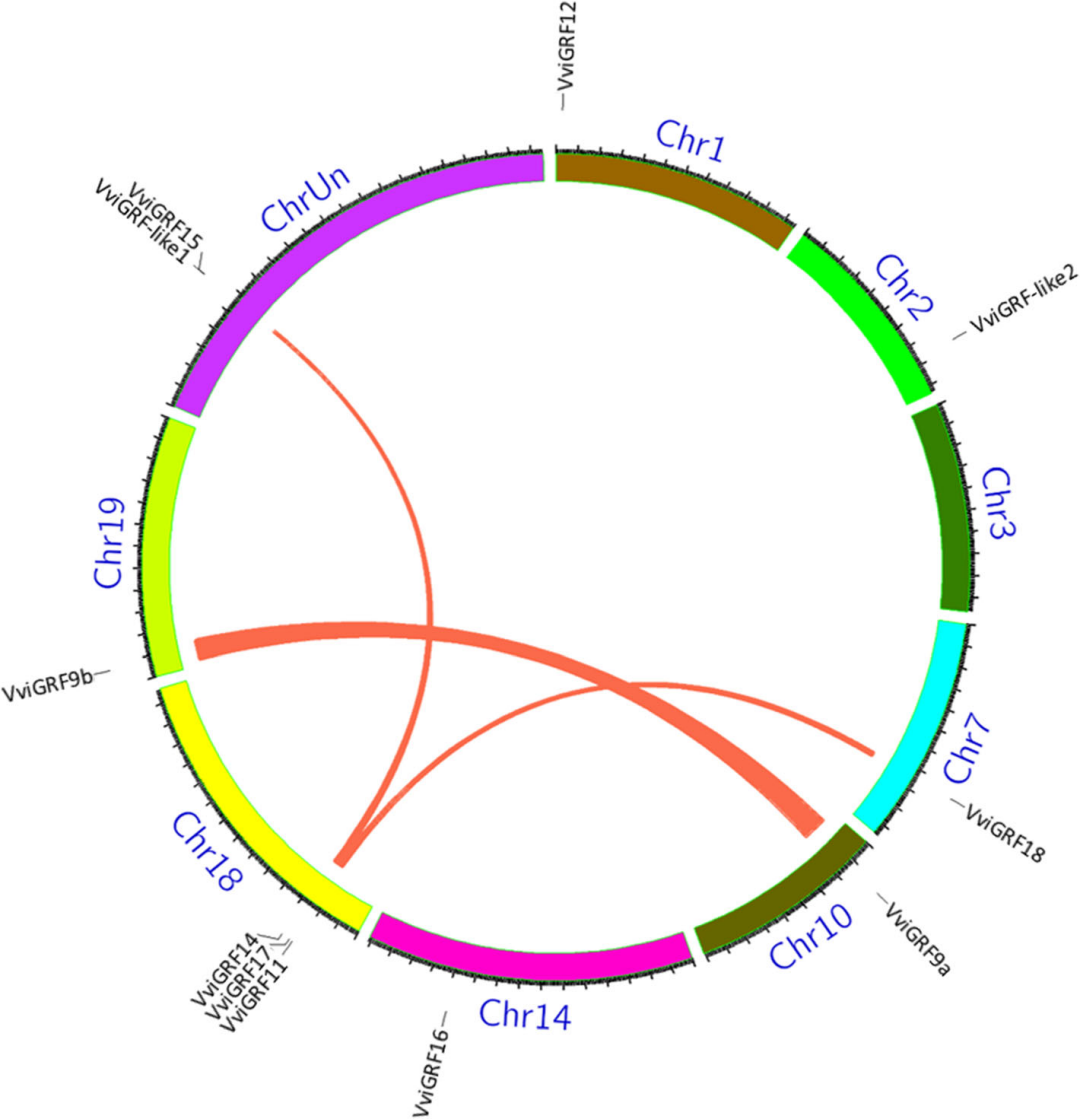
The synteny analysis of 14–3-3 family in grape. Only chromosomes contained *VviGRF* genes are represented in a circle in this figure. Chromosomes were drawn in different colours. The approximate location of *VviGRF* genes is shown by short black lines on the circle. Red curves linking *VviGRF* genes represent the duplication events occurred in grape 14–3-3 gene family

The location data of *VviGRF*s were also obtained from the genome annotation files (http://genomes.cribi.utnipd.it/DATA/). The gene location map was constructed using MapChart (Fig. 4) [31]. Moreover, the gene duplication landscape was obtained using the MCScanX [32]. Each duplicate segment with *VviGRF* genes was selected, and the syntonic map was generated using CIRCOS [33]. The putative duplicated genes were linked by the connection lines (Fig. 5).

### Transcriptomic resources

In this work, published data GSE36128, GSE62744, SRP018199 and SRP091989 were used for the analysis of expression profiles of *VviGRF* genes in 54 tissues and organs, during berry ripening, and in cold and heat stress responses, respectively [34–37]. To survey the temporal and tissue-specific expression of *VviGRF*s, 54 tissues and organs in *V. vinifera* cv Corvina were collected in GSE36128 (Additional file 2, Fig. 6). To search the expression pattern of *VviGRF*s in different varieties during berry development, berries at four growth periods (Pea, Touch, Soft, Harvest) in five grape varieties, namely, Sangiovese, Barbera, Negro amaro, Refosco and Primitivo were used in GSE62744 (Fig. 7a). In SRP018199, the *V. vinifera* cv. Muscat Hamburg seedlings were used under the gradual cooling treatment (decreased at 5 °C per hour from 24 °C to 4 °C and then 4 °C for additional 4 h) to analyse the expression profile of *VviGRF*s in response to cold treatment. Besides, to survey their response to heat treatment, detached leaves in *V. vinifera* cv ‘Jingxiangyu’ plantlets were treated under 25 °C, 35 °C, 40 °C and 45 °C.

**Fig. 6:**
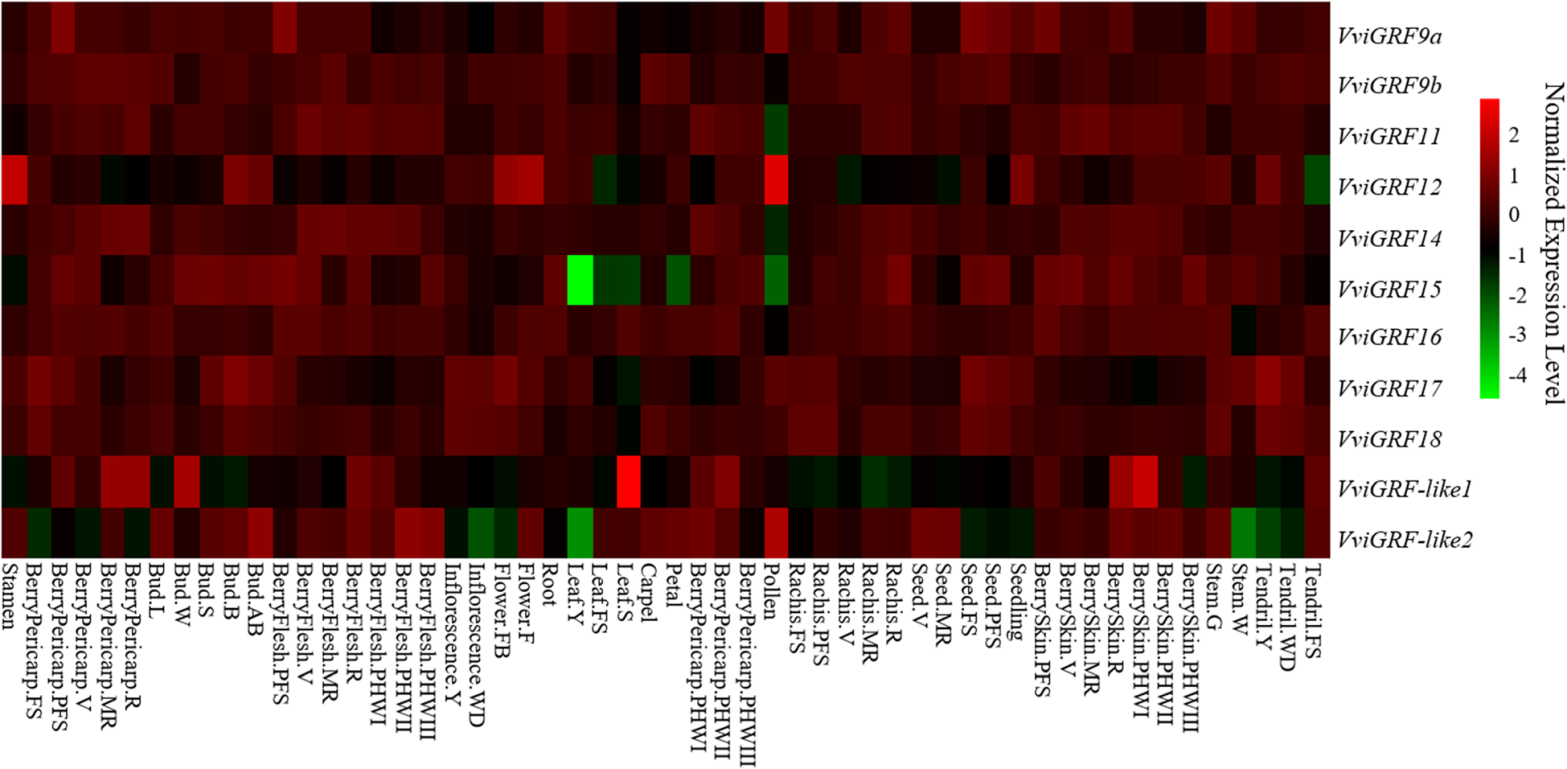
The temporal and tissue-specific expression of *VviGRF* genes based on the GSE36128 [35]. Expression data were processed with log2 normalization. The horizontal axis shows fifty-four tissues and organs, which can be seen in Additional file 2. The colour scale represents relative expression levels

**Fig. 7:**
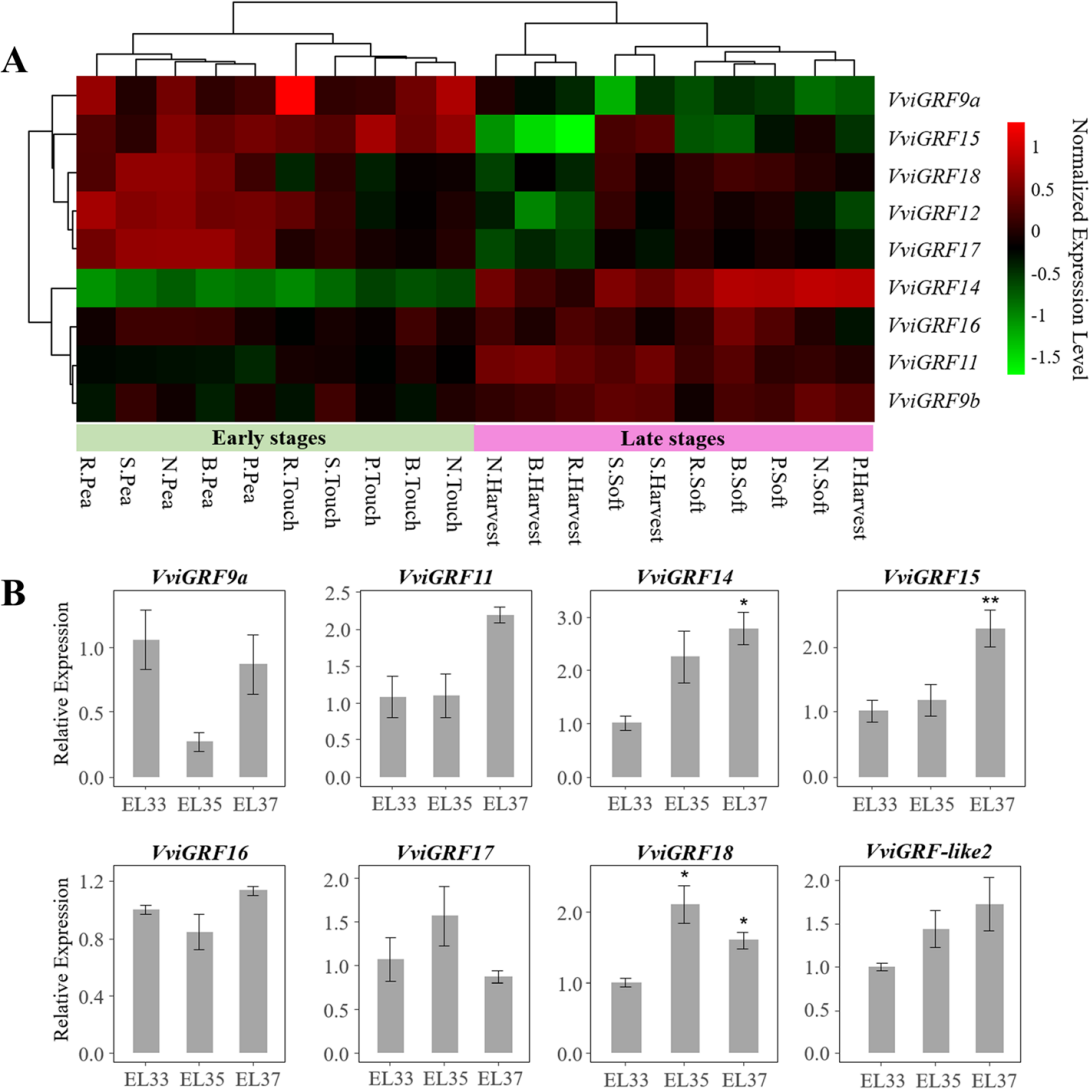
Expression profile of *VviGRF*s during grape berry ripening. (**a**) Expression pattern of *VviGRF*s in five grape varieties during berry developmental stages. The used grape berries were taken from five red-skin grape varieties, namely, Sangiovese (S.), Barbera (B.), Negro amaro (N.), Refosco (R.), and Primitivo (P.). Berries were collected at four phenological stages, as follows: pea-sized berries at 20 days after flowering (Pea), berries beginning to touch just prior to veraison (Touch), softening berries at the end of veraison (Soft), and berries ripe for harvest (Harv) [36]. The obtained data were processed using log2 normalization. The colour scale represents relative expression levels. (**b**) qRT-PCR results of eight *VviGRF* genes during berry ripening. As previous study indicated [38], EL33, EL35 and EL37 represent three important stages during berry ripening, respectively. EL33 means the stage when berries are still hard and green, EL35 means the veraison when berries begin to colour and enlarge, and EL37 presents the period when berries will mature soon but not quite ripe. Pea, touch, soft and harvest stages in **a** correspond to EL31, EL34, EL37 and EL38, respectively. Period before veraison is named the early stage in this study, and the period after veraion is the late stage. Thus EL33, pea, touch belong to the early stages, and EL37, soft, harvest belong to the late stage. Expression data was normalized to *VviActin* gene expression level, and every VviGRFs at EL33 stage was normalized as “1”. The mean expression value was calculated from three replications. Vertical bars indicate the standard error of mean. **P < 0.01 and *P < 0.05 compared with expression level at EL33 stage

### Plant material

To verify exon—intron structure, specific cDNA and DNA sequences were obtained from three biological replicates of green stem in *V. vinifera* cv. ‘Cabernet Sauvignon’. To analyse the *14–3-3* expression pattern in grape during berry ripening, three biological replicates represented by 15—20 grape berries from *V. vinifera* cv. ‘Cabernet Sauvignon’ were sampled at three developmental stages (EL33, EL35 and EL37 [38]). The grapevines were planted in south-to-north oriented rows by similar management conditions at the Germplasm Repository for Grapevines, Institute of Botany, the Chinese Academy of Sciences, Beijing, China (39° 54’ N, 116° 23′ E).

To analyse the *14–3-3* expression in grape under heat stress, 30-day-old leaves taken from *V*. *davidii* were heated in a water bath (25 °C for 2 h and 47 °C for 40 min) with three biological replicates. *V*. *davidii* were planted in the same condition as *V. vinifera* cv. ‘Cabernet Sauvignon’ described above. Heat treatment was processed according to a previous study [39]: leaf discs (5.5 cm in diameter) cut from the detached leaves were wrapped in a wet paper and placed in a small vessel made of aluminium foil. Leaf discs were sampled when vessels floated in a temperature-controlled water bath at 25 °C for 2 h and subsequently at 47 °C for 40 min. The control plants were treated in the same condition as that in heat treatment, except for temperature controlled at 25 °C.

To study the expression profile of *VviGRF*s in response to cold stress, six-week-old *Vitis amurensis* plantlets grown on 1/2 Murashige and Skoog (pH 5.8, 0.7% agar and 1% sucrose in 120 mL conical flasks) solid medium were placed in 4 °C (cold treatment) and 24 °C (control) chambers with a 16 h light/8 h dark cycle and 100 μmol m^− 2^ s^− 1^ light intensity. Shoots with the first fully expanded leaf were sampled after 0 h, 8 h and 12 h cold treatment with three biological replicates.

### RNA extraction and qRT-PCR analysis

All samples were collected then preserved in liquid nitrogen immediately, and stored at − 80 °C for RNA and DNA extraction. Total RNA and DNA were extracted using the RNAprep Pure Plant Kit (DP432, Tiangen Biotech, Beijing, China) and Plant Genomic DNA Kit (DP305–02, Tiangen Biotech, Beijing, China) respectively, according to the manufacturer’s instructions. The cDNA used for verifying exon—intron structure was synthesised using HiScript® II 1st Strand cDNA Synthesis Kit (R211–01, Vazyme Biotech Co., Nanjing, China). To amplify the complete cDNA and DNA fragments corresponding to the *VviGRF* genes, specific primers for each gene were designed through NCBI Primer-BLAST (http://www.ncbi.nlm.nih.gov/tools/primer-blast/, Additional file 3), and the PCR reactions were performed using PrimeSTAR Max DNA Polymerase (R045A, Takara Biotechnology Co., Ltd., Dalian, China) with the following thermal cycling profile: 98 °C for 3 min, 34 cycles of 98 °C for 10 s and 60 °C for 5 s and 72 °C for 50 s, 72 °C for 5 min, 12 °C for ∞. PCR products were submit to Beijing Majorbio Company, and the sequencing results can be seen in Additional file 4. The cDNA used for qRT-PCR analysis was synthesised through reverse transcription of obtained total RNA using HiScript® II Reverse Transcriptase (R223–01, Vazyme Biotech Co., Nanjing, China). The synthesised cDNA was subjected to qRT-PCR with an Opticon thermocycler (CFX Connect Real-Time System; Bio-Rad, Hercules, CA) using SYBR Green PCR master mix (Vazyme, Nanjing, China) according to the manufacturer’s instructions. In qRT-PCR, *VviActin* (Accession: EC969944) was used as reference gene, and specific primers for *VviGRF* genes were designed by using Primer-BLAST (Additional file 5). The specificity of designed primers was verified through gel electrophoresis and sequencing. PCR reactions were as follows: 95 °C for 10 min, 40 cycles of 95 °C for 10 s and 60 °C for 30 s. Each sample was prepared in three biological and technical replicates. The relative expression levels of the *VviGRF*s were calculated using the 2^−ΔΔCt^ method. Statistical difference was determined by t-test (**P < 0.01, *P < 0.05, *n* = 3) using R program [40].

## Results

### Identification, multiple sequences alignment, and phylogenetic analysis of grape 14–3-3 family

Nineteen 14–3-3 proteins were obtained by using HMM and BLASTP. A total of 11 proteins containing 14–3-3 domain were identified in grape after searching in NCBI-CDD. The results of sequence alignment showed that VviGRFs contained several conserved domains in their aligned sequences, and nine α helices were identified in their secondary structures (Fig. 1). Based on phylogenetic results, these proteins were classified into two groups, namely, epsilon (VviGRF9a/9b/11/12) and non-epsilon groups (VviGRF14/15/16/17/18/−like1/−like2) (Figs. 2 and 3a). VviGRF proteins contained 64 (VviGRF-like2) to 582 (VviGRF-like1) amino acid residues, their Mol wts varied from 6.91 kDa (VviGRF-like2) to 66.03 kDa (VviGRF-like1), and their PIs ranged from 4.74 (VviGRF16) to 7.57 (VviGRF-like2) (Table 1).

### Gene structure and motif analysis

Figure 3b illustrates the predicted motif and gene structure of *VviGRF* genes. And the exon—intron structure of *VviGRF* genes have been verified by sequencing, which was accordant with our predicted analysis (Additional file 4). The number of exon in *VviGRF*s varied from one (*VviGRF-like2*) to seven (*VviGRF9b*/ *11*/ *12*), and nine out of 11 *VviGRF*s showed not less than four exons (except for *VviGRF-like1* and *VviGRF-like2*). A search on MEME program identified seven motifs in VviGRF proteins. The number of motifs in VviGRFs ranged from 1 to 7, and the length of motifs varied from 8 (motif 6) to 50 (motifs 1–3) amino acids. Most VviGRFs contained all seven motifs, except for VviGRF-like1 and VviGRF-like2. VviGRF-like1 contained five motifs without motifs 2 and 5. VviGRF-like2 only had motif 7. Moreover, NCBI-CDD results showed that the 14–3-3 domain of VviGRF-like2 was incomplete in N-terminal, VviGRF-like1 was incomplete in C-terminal. Furthermore, VviGRF-like1 contained a 14–3-3 domain, a domain of unknown function (DUF4283) and a zinc-binding motif (Additional file 6). Associated with gene and motif structure, the position of introns was conserved in grape 14–3-3 family (Fig. 3). In both groups, motif 1 and 6 were translated by a single exon, motif 2 and motif 5 were translated from sequences modelled by “exon-intron-exon”. In epsilon group, motifs 3, 4 and 7 in all four members were translated by “exon-intron-exon” sequences. In non-epsilon group, the sequence of 6 members (except for VviGRF-like2) encoding motif 7 also shown in this model. The detail information of introns position in “exon-intron-exon” sequences was shown in Additional file 7. Between epsilon group and non-epsilon group, the position of introns was absolutely different. But in the same group, the introns were present at the same position within their encoding sequences.

### Chromosome distribution and synteny analysis of *VviGRF*s

Eleven *VviGRF* genes were distributed unevenly on seven chromosomes and chrUn in grape (Table 1 and Fig. 4). The chr18 contained three *VviGRF* genes (*VviGRF11*, *VviGRF14* and *VviGRF17*), chr1, 2, 7, 10, 14 and 19 only contained one *VviGRF* gene. *VviGRF15* and *VviGRF-like1* were located on chrUn. We also conducted a synteny analysis on *VviGRF* genes to investigate the duplication event occurring in the grape 14–3-3 family (Fig. 5). Three duplication events were observed between chr10 and chr19 (*VviGRF9a* and *VviGRF9b*), chr18 and chrUn (*VviGRF14* and *VviGRF-like1*), chr7 and chr18 (*VviGRF17* and *VviGRF18*), which evolved from segment duplication.

### Tissue-specific expression of *VviGRF* genes

To analyse the temporal and tissue-specific expression of *VviGRF*s in grapevine, we assessed their expression data in 54 tissues and organs obtained from the published GSE36128 (Fig. 6). Most *VviGRF*s exhibited a quite moderate expression in different tissues and organs, but some genes showed very high or very low expression in certain tissues. For instance, *VviGRF12* was normally lowly expressed in most tissues and organs, but not in some floral organs such as stamen, bud, flower and pollen. And in normal conditions, *VviGRF15* showed a high expression level in most tissues and organs, except for leaf, petal and pollen. *VviGRF-like1* was generally lowly expressed in most tissues and organs, except for senescencing leaf, bud, berry pericarp and berry skin. *VviGRF-like2* exhibited a high expression level in most tissues, but lowly expressed in well-developed inflorescence, young leaf, and woody stem.

### Expression pattern of *VviGRFs* during berry development

To determine the *VviGRF* expression in different grape genotypes, we analysed their expression levels in five grape varieties during berry ripening (GSE62744, Fig. 7a). Except that *VviGRF*s in Sangiovese showed similar expression at soft and harvest stages, the expression patterns of *VviGRF*s in five varieties were similar at the early (pea and touch) and late (soft and harvest) stages of ripening. *VviGRF9b*, *VviGRF11*, *VviGRF14* and *VviGRF16* exhibited low expression level at the early stages and high expression level at the late stages. By contrast, *VviGRF9a* and *VviGRF15* showed high and low expression levels at the early and late stages, respectively. The expression level of *VviGRF12*, *VviGRF17* and *VviGRF18* was constantly changed during berry ripening. At early stages, these three genes expressed highly at pea stage and lowly at touch stage, whereas at late stages, they expressed highly at soft stage and lowly at harvest stage. To study their expression patterns during grape berry development (Fig. 7b), eight *VviGRF*s in *V. vinifera* cv. ‘Cabernet Sauvignon’ were selected to qRT-PCR. Five out of 8 *VviGRF*s showed no significant expression level at three key stages during berry ripening (EL33, EL35 and EL37). Only *VviGRF18* expression was up-regulated at both EL35 and EL37 stages. *VviGRF14* and *VviGRF15* were only up-regulated at EL37.

### Response to cold and heat stresses

**Fig. 8:**
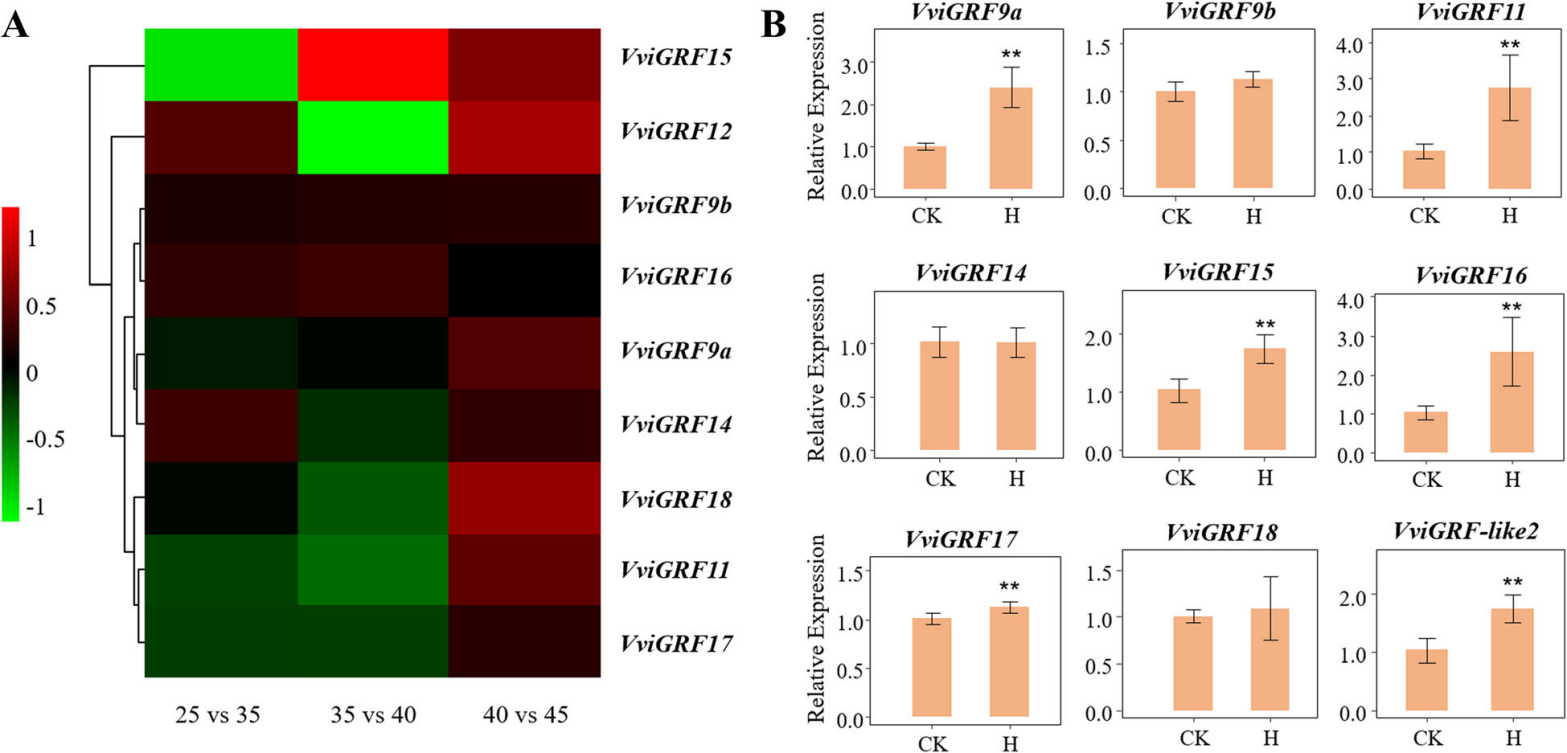
Expression pattern of *VviGRF*s under heat stress. (**a**) Expression profile of *VviGRF* genes in response to heat treatment on published data RNA-seq SRP091989 [37]. Log2 change fold between two treatment temperatures was used to present changed expression level under heat treatment by R software. For example, the expression level of 25 vs 35 was calculated from the formula: log2 (the expression at 35 °C / the expression at 25 °C). The colour scale represents relative expression levels. (**b**) qRT-PCR profiles of *VviGRF* genes by heat treatment. CK means control, H means heat treatment. Expression level of heat stress was normalized to *VviActin* gene expression level, and every *VviGRF*s at CK was normalized as “1”. The mean expression value was calculated from three replications. Vertical bars indicate the standard error of mean. **P < 0.01 compared with expression level at CK

RNA-seq GSE89113 was used to investigate the response of *VviGRFs* to heat stress (Fig. 8a). Four *VviGRF*s (*VviGRF9b*/*12*/*14*/*16*) indicated up-regulated expression in detached leaves under 35 °C treatment compared with that at 25 °C, whereas the expression level of three *VviGRF*s (*VviGRF11*/*15*/*17*) showed down-regulation. Among them, *VviGRF15* exhibited a significantly down-regulated expression (log_2_ fold change = − 0.969124). Under 40 °C, the expression levels of five members (*VviGRF11*/*12*/*14*/*17*/*18*) and three members (*VviGRF9b*/*15*/*16*) were down-regulated and up-regulated at 35 °C, respectively. Among them, *VviGRF12* showed significantly decreased expression, but *VviGRF15* significantly increased (|log_2_ fold change| > 1). Under 45 °C, all of nine *VviGRF*s showed an increase in expression compared with that at 40 °C. According to the data published in SRP018199, *GSVIVT01012207001* and *GSVIVT01009141001* (corresponding *VviGRF12* and *VviGRF14* in 12X V1), the *V. vinifera* cv. Muscat Hamburg seedlings showed up-regulated expression in response to the gradual cooling treatment (decreased at 5 °C per hour from 24 °C to 4 °C and subsequently 4 °C for additional 4 h, Additional file 8).

**Fig. 9:**
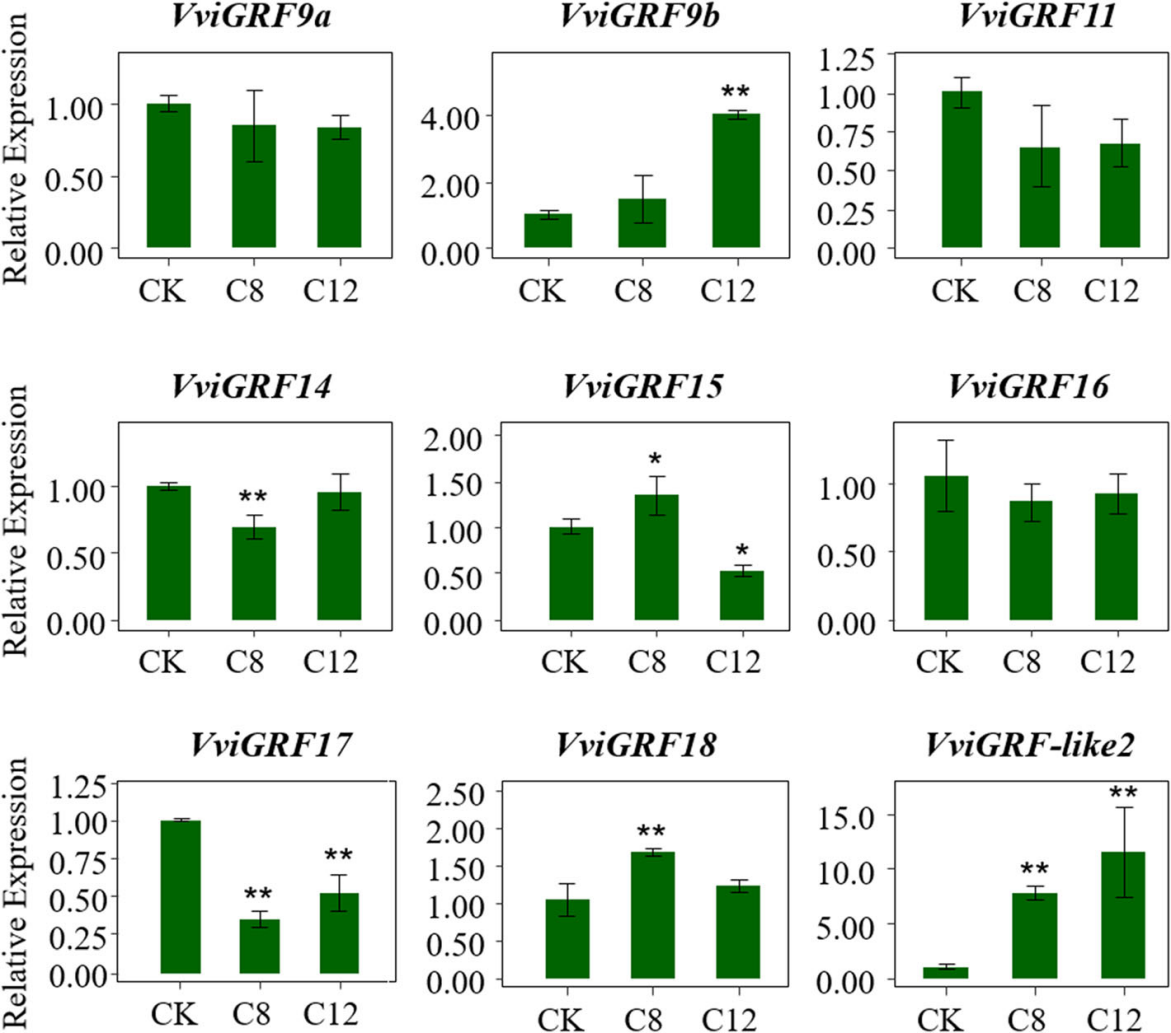
qRT-PCR profiles of *VviGRF* genes under cold treatment. CK means control, C8 and C12 represent 8 h and 12 h 4 °C treatment, respectively. Expression data of cold stress was normalized to *VviActin* gene expression level, and every VviGRFs at CK was normalized as “1”. The mean expression value was calculated from three replications. Vertical bars indicate the standard error of mean. **P < 0.01 and *P < 0.05 compared with expression level at CK

To obtain further understanding about the putative function of *VviGRF*s under heat and cold stresses, the expression patterns of nine *VviGRF*s in *V*. *davidii* and *V. amurensis* were analysed by qRT-PCR (Figs. 8b and 9). Three *VviGRF*s (*VdGRF9b*/*14*/*18*) showed no significant change in expression under heat treatment (25 °C 2 h and 47 °C 40 min), whereas the expression level of six *VviGRF*s (*VdGRF9*a/*11*/*15*/*16*/*17*/−*like2*) were up-regulated (Fig. 8b). After cold treatment (4 °C, 0–12 h, Fig. 9), except for three *VviGRF*s (*VaGRF9a*/*11*/*16*), six *VviGRF*s demonstrated significant expression change. *VaGRF17* and *VaGRF*-*like2* continuously decreased and increased during cold treatment, respectively. The expression of *VaGRF9b* exhibited no significant change at 8 h but increased at 12 h. *VaGRF15* and *VaGRF18* increased at 8 h and subsequently decreased at 12 h. *VaGRF14* decreased at 8 h and subsequently increased at 12 h.

## Discussion

In this work, we identified and characterized the 14–3-3 gene family in *V. vinifera*. It is composed by 11 members, a number higher than in rice [11], but lower respect to *Arabidopsis* [16], tomato [12], cotton [13], banana [14], switchgrass [41], and soybean [15]. Based on amino acid sequences, gene structure and phylogenetic relationship with *A. thaliana*, the 11 VviGRF proteins were classified into two groups (Table 1 and Fig. 2, Fig. 3a). This result is also in accordance with previous studies on other species [11, 12, 42]. For example, five and eight AtGRF proteins were contained in epsilon and non-epsilon groups in *Arabidopsis*, respectively.

A total of seven motifs were found in the grape 14–3-3 family. Epsilon group members contained all seven motifs, whereas non-epsilon group members commonly possessed less motifs than those of epsilon group members (e.g., VviGRF-like1 and VviGRF-like2). Similar to the motif analysis, epsilon group members commonly possessed more exons or introns than those of non-epsilon group, which also verified by RT-PCR (Table 1, Fig. 3b, and Additional file 4). Generally, only epsilon group members contained introns within sequences encoding motifs 3 and 4.This observation revealed that different groups of phylogenetic tree presented different motifs and exon—intron structures. This finding can also be observed in other species, such as *Arabidopsis* and rice; epsilon group members often present two extra introns in the N- or C-terminal compared with those of non-epsilon group [11, 42]. Take *Arabidopsis* for example (Additional file 1), there were a total of six motifs, and both their sequences and distribution were similar with those in grapevine. And as found in grapevine, sequences encoding motifs in *Arabidopsis* 14–3-3 family also possessed “exon-intron-exon” model. Moreover, Additional file 9 showed that the position of introns in this model was conserved in the same phylogenetic group, which was in accordance with grapevine. And between *Vitis* and *Arabidopsis*, introns within motifs 2 and 4 located in the same position. These similarities suggested different distribution of “exon-intron-exon” sequences encoding motifs between epsilon and non-epsilon groups, and also revealed the conservation of the exon—intron and motif structure in 14–3-3 family.

In grape 14–3-3 family, VviGRF-like1 was the longest protein, which displayed a low homology with other 14–3-3 members. And different from other VviGRFs, VviGRF-like1 contained an additional unknown function (DUF4283) and a zinc-binding motif. DUF4283 contains two highly conserved tryptophan residues and possibly provides binding/guiding region for diverse range of other domains (view pfam14111 in NCBI-CDD) [24]. Moreover, NCBI-CDD results (Additional file 6) showed that the 14–3-3 domain of VviGRF-like2 lacked motif 1/3/4/6, and it was incomplete in N-terminal; the 14–3-3 domain of VviGRF-like1 lacked motif 2/5, and it was incomplete in C-terminal. These results indicated that motifs 1/3/4/6 and 2/5 may be the necessary components for the N- and C-terminals of 14–3-3 domain, respectively.

Gene duplication events are crucial in genomic rearrangement and often result in the expansion of gene family, which includes tandem, segment and transposition duplication [43]. In grape 14–3-3 family, three pairs of genes evolved from segment duplication, but no tandem duplication event occurred (Fig. 5), indicating that the segment duplication may be the dominant gene duplication on the expansion of this family. And as for the remaining five *VviGRF*s, they may evolve in an early divergence time or be obtained from gene transposition.

To further understand the putative function of *VviGRF*s, the expression pattern of *VviGRF*s was searched for reference in the Vespucci database [44], the RNA-seq and expression-array data were also analysed. Fig. 6 reveals that *VviGRF*s were expressed in all investigated tissues and organs, and several *VviGRF*s showed tissue-specific expression in different tissues (e.g., *VviGRF12*/*15*/−*like1*/−*like2*). This phenomenon is also supported by some *14–3-3* genes in switchgrass, tomato, *Arabidopsis* and barley microspore embryogenesis [41, 45–47].

During berry ripening, *VviGRF*s in Sangiovese expressed similar pattern at soft and harvest stages, which may be caused by the varietal character or close sampling time at these two stages. Except this, *VviGRF*s in different varieties exhibited similar expression patterns between the early and late stages of berry ripening. In accordance with the results, *VviGRF14*, *VviGRF15* and *VviGRF18* displayed significant change in expression levels during EL33 (early stage), EL35, and EL37 stages (late stage). However, the up-regulated or down-regulated tendency of *VviGRF15* differed in Fig. 7a and b, which may be impacted of variety difference. Taken together, some *VviGRF* genes presented changed expression during berry ripening, and they may be involved in the regulation of grape development. Accordingly, previous studies also demonstrated that plant 14–3-3 proteins are involved in fruit development and ripening processes. For example, most *MaGRF* transcriptions are significantly accumulated during fruit development and postharvest ripening in banana [14]. Transcription profiles of switchgrass suggested that four *PvGRF*s may be involved in regulating lignin metabolism, and *PvGRFr* may participate in flower development [41]. In *Arabidopsis*, RNA interference revealed the fundamental role of epsilon group members in regulating PIN polarity and plant development [48].

In the process of heating-up temperature (RNA-seq SRP091989, Fig. 8a), the expression of *VdGRFs* changed significantly, and nine genes presented up-regulated expression at 45 °C. In accordance with this result, six *VdGRFs* exhibited up-regulated expression in response to heat stress (Fig. 8b). Similar to this result, most *14–3-3* genes also display up-regulated expression under heat treatment in rice and *Brachypodium distachyon* L. [11, 49]. In *V. amurensis*, six *VaGRF* expression levels significantly changed in response to cold treatment, and five of six *VaGRF*s up-regulated or down-regulated after 8 h treatment, thereby revealing their potential roles in cold stress response (Fig. 9). In accordance with previous studies [11, 50, 51], two *AtGRF*s, five *OsGRF*s and two *TaGRF*s also indicated that cold stress induced significant expression changes in *Arabidopsis*, rice and wheat, respectively. *VviGRF15*, *VviGRF-like2* and *VviGRF17* showed significantly change in expression level under both cold and heat treatments, which indicated their potential roles in multiple-abiotic-stress responses. In summary, *VviGRF*s were important genes involved in cold and heat stress responses.

## Conclusions

In grape, a total of 11 *VviGRF*s were identified and classified into epsilon and non-epsilon groups according to their phylogenetic relationship with *A. thaliana*. Protein and gene structure, together with gene duplication event analyses were performed to determine the characteristics and potential functions of grape 14–3-3 family. Published transcription data and qRT-PCR were analysed to gain further understanding regarding the putative function of *VviGRF* genes. The expression level of three *VviGRF*s showed significantly changes during berry ripening. Six *VviGRF*s expression altered under abiotic stress. The present results revealed the potential roles of *VviGRF* genes in berry development and abiotic stress response. This work provides new insight into grape 14–3-3 family and a basis for further study on its functional mechanism.

## Additional files

Additional file 1: Phylogenetic relationship, motif and gene structure analysis of AtGRF proteins. (**A**) Phylogenetic tree of 14–3-3 family in *Arabidopsis*. (**B**) Gene structure and motif analysis of 14–3-3 members in *Arabidopsis*. The black blocks represent the untranslated region (UTR), the gray blocks represent exons, and the black lines represent introns. (**C**) The amino acid sequences of motifs in AtGRF proteins. Arrows showed the intron position appeared in “exon-intron-exon” sequences. Red arrow means the epsilon group, green arrow means the non-epsilon group. The detail information for the intron position was shown in Additional file 9. (TIF 2369 kb)

Additional file 2: The organs presented in Fig. 6 from published data GSE36128. (DOC 63 kb)

Additional file 3: The primer sequence for verifying exon—intron structure of seven *VviGRF* genes. (DOC 30 kb)

Additional file 4: Comparison of sequencing results and exon—intron structure of seven *VviGRF* genes. (**A**) cDNA Sequence alignment of *VviGRF*s. ‘_sequencing’ means cDNA of each *VviGRF* cloned from green stem in Cabernet Sauvignon. ‘_cDNA’ means reference sequences of each *VviGRF* cDNA in Point Noir. (**B**) DNA Sequence alignment of *VviGRF*s. ‘_sequencing’ means DNA of each *VviGRF* cloned from green stem in Cabernet Sauvignon. ‘_DNA’ means reference sequences of each *VviGRF* DNA in Point Noir. Sequence alignment analysis was performed by using DNAMAN version7. Sequences of seven *VviGRF*s in Cabernet Sauvignon were homologous with their reference sequence in Point Noir (Identity > 94%). (**C**) The exon—intron structure of seven *VviGRF*s. CS means sequencing gene structure in Cabernet Sauvignon, PN means reference gene structure in Point Noir. (DOC 18109 kb)

Additional file 5: The primer sequence for qRT-PCR. (DOC 31 kb)

Additional file 6: The conserved domain analysis of VviGRF proteins. 14–3-3 domain was present in blue strips, and the jagged edges means incomplete N- or C-terminal. (TIF 679 kb)

Additional file 7: Motif sequences of VviGRF proteins. Black arrow shows the introns position appeared in “exon-intron-exon” sequences. (TIF 5120 kb)

Additional file 8: Expression profile of *14–3-3* genes in *V. vinifera* cv. Muscat of Hamburg under cold stress from published data SRP018199. (DOC 28 kb)

Additional file 9: Motif sequences of AtGRF proteins. Black arrow shows the introns position appeared in “exon-intron-exon” sequences. (TIF 6446 kb)

Additional file 10: Detail information of grape 14–3-3 family. (DOC 39 kb)

## Abbreviations

CDD: Conserved Domain Data
chr: Chromosome
ChrUn: Unanchored chromosome
DEAE: Diethyl-aminoethanol
GSDS: Gene Structure Display Server
H^+^-ATPase: Plasma membrane proton pumping
HMM: Hidden Markov Model
Mol wt: Molecular weight
PI: Isoelectric point
UTR: Untranslated region
