# Supplementary figures and images for: Genome-wide identification and characterization of the 14–3-3 family in Vitis vinifera L. during berry development and cold- and heat-stress response
Source: BMC Genomics. 2018 Aug 2;19:579. doi: 10.1186/s12864-018-4955-8 (PMC6090852; doi:10.1186/s12864-018-4955-8)

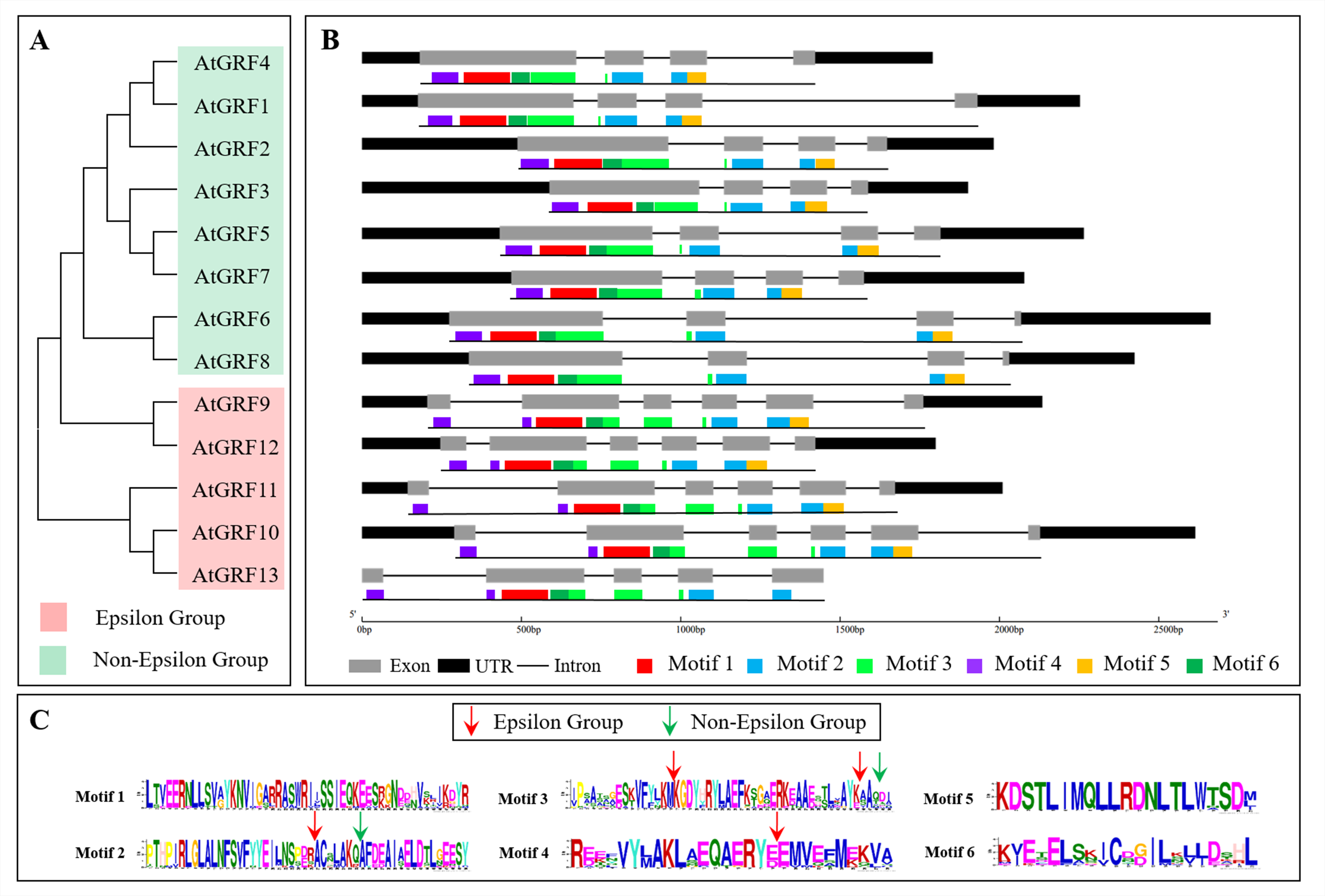

Supplement: Supplementary file 1 — Phylogenetic relationship, motif and gene structure analysis of AtGRF proteins. (A) Phylogenetic tree of 14–3-3 family in Arabidopsis. (B) Gene structure and motif analysis of 14–3-3 members in Arabidopsis. The black blocks represent the untranslated region (UTR), the gray blocks represent exons, and the black lines represent introns. (C) The amino acid sequences of motifs in AtGRF proteins. Arrows showed the intron position appeared in “exon-intron-exon” sequences. Red arrow means the epsilon group, green arrow means the non-epsilon group. The detail information for the intron position was shown in Additional file 9. (TIF 2369 kb) [file 12864_2018_4955_MOESM1_ESM.tif]

**
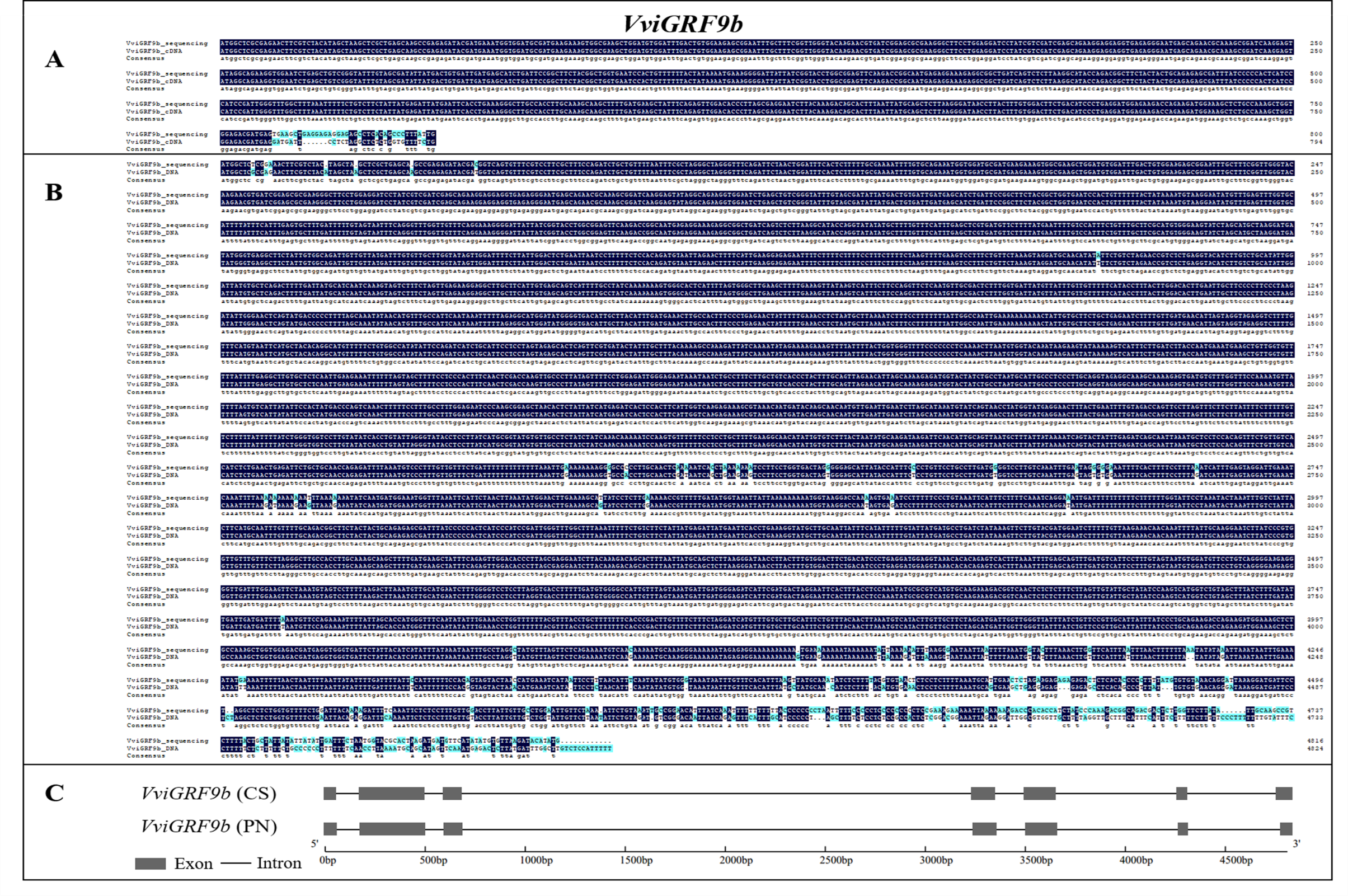
**

**
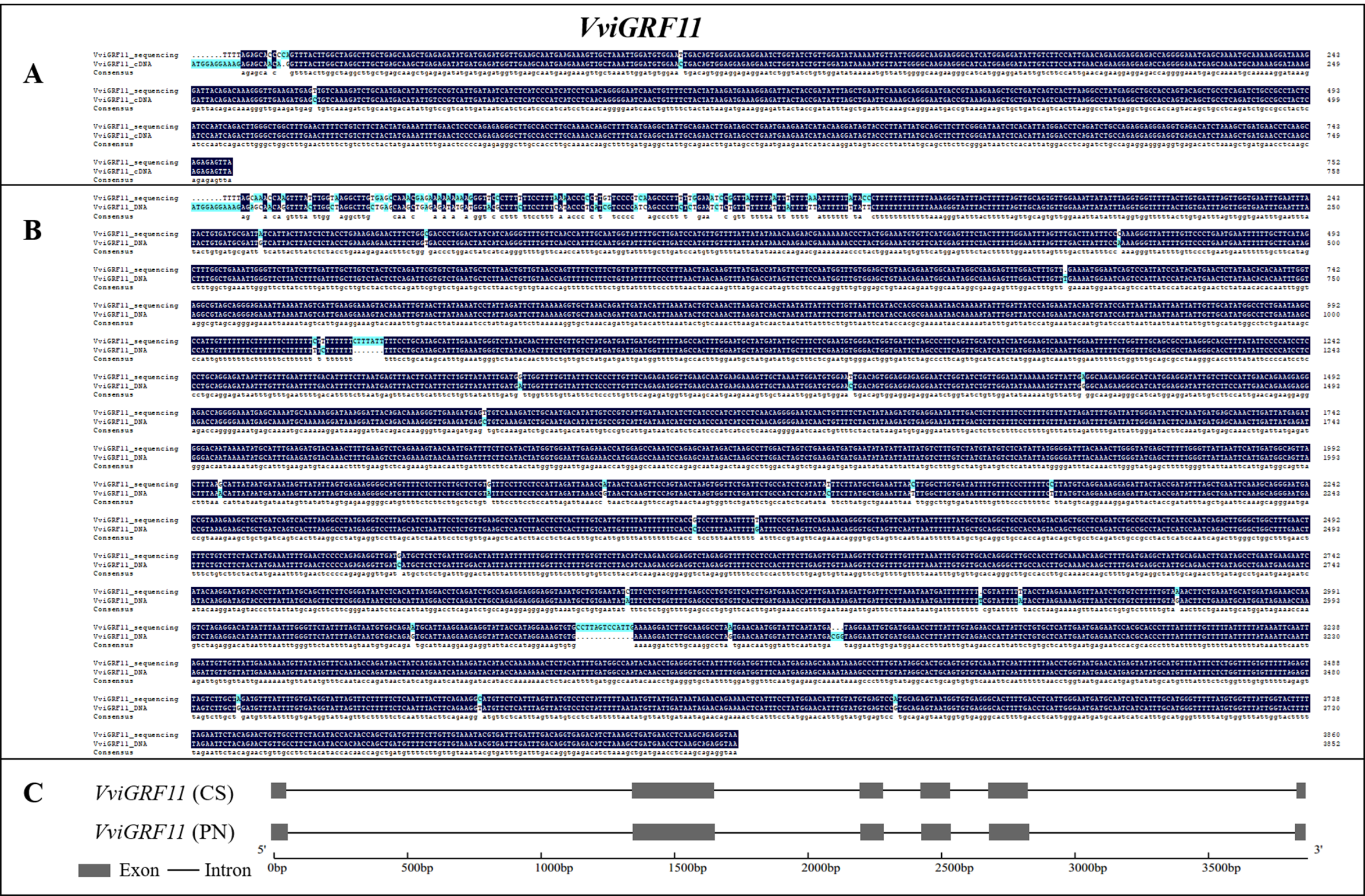
**

**
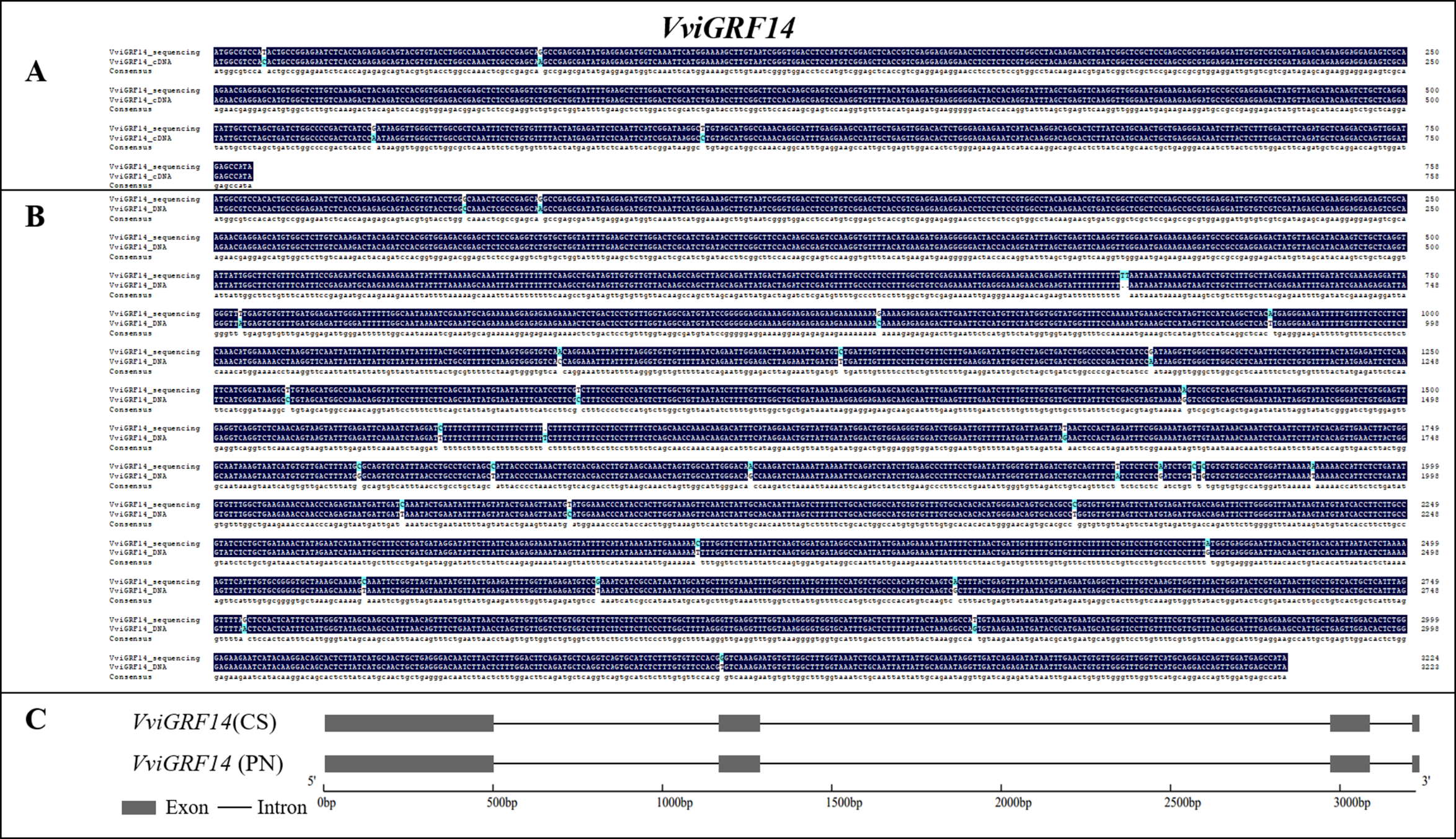
**

**
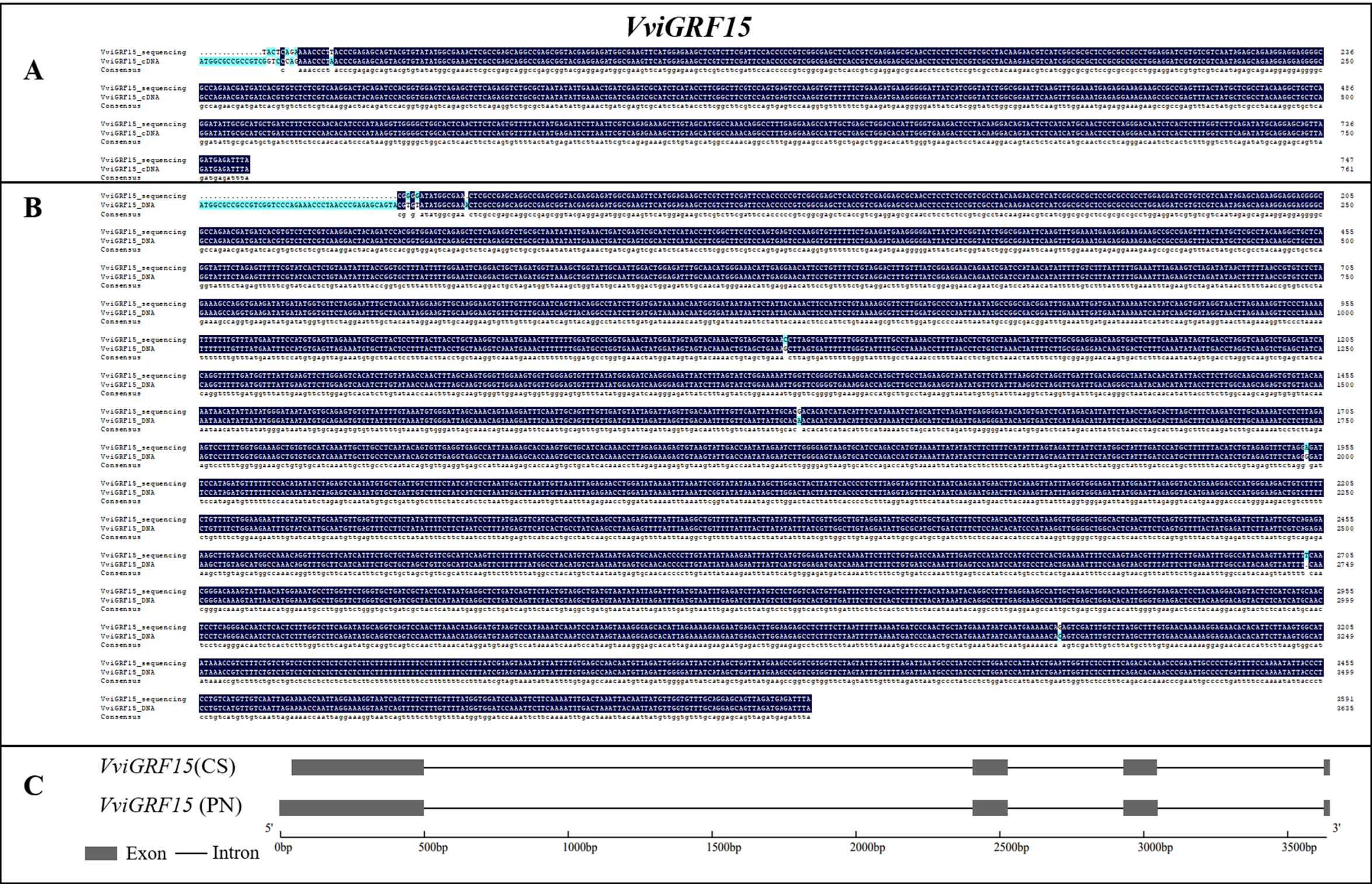
**

**
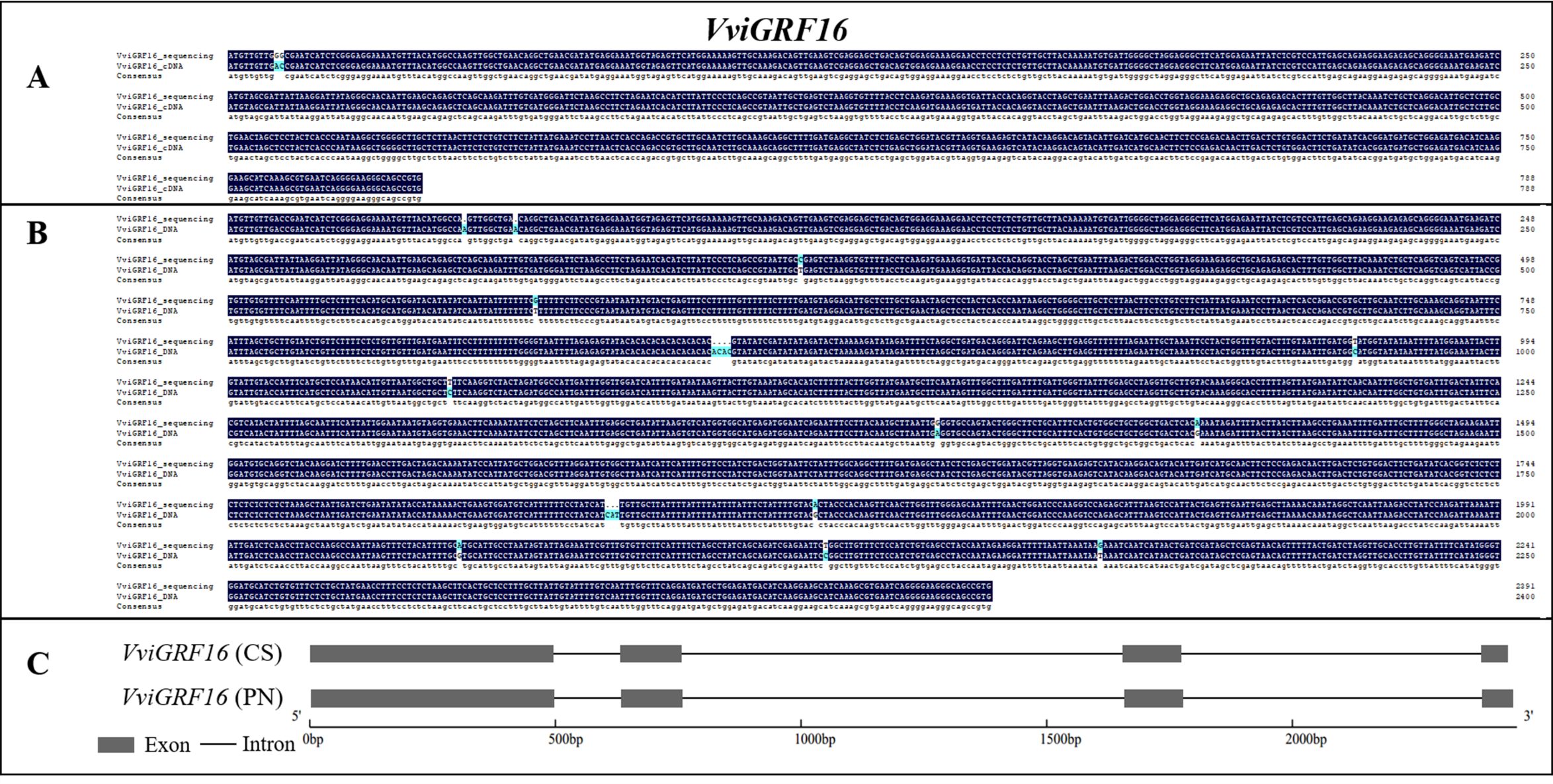
**

**
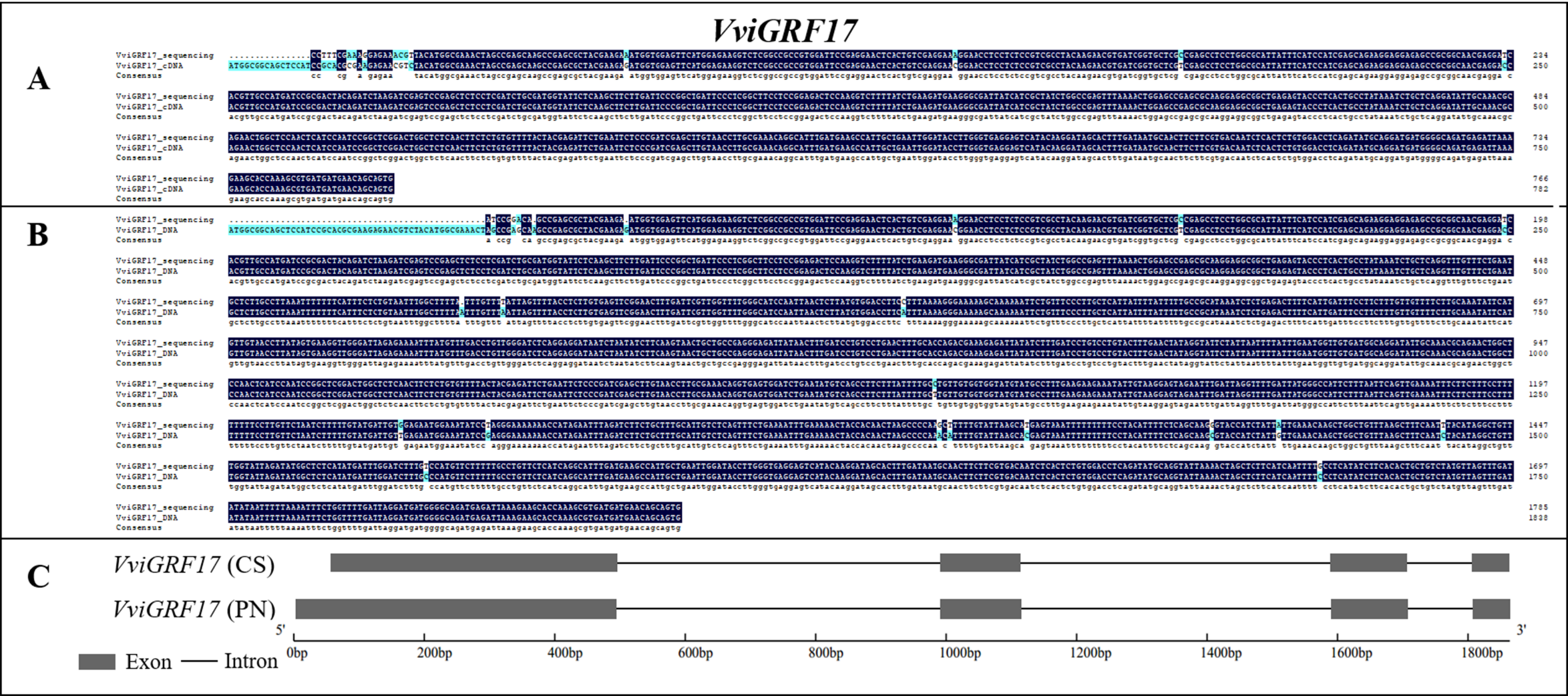
**

**
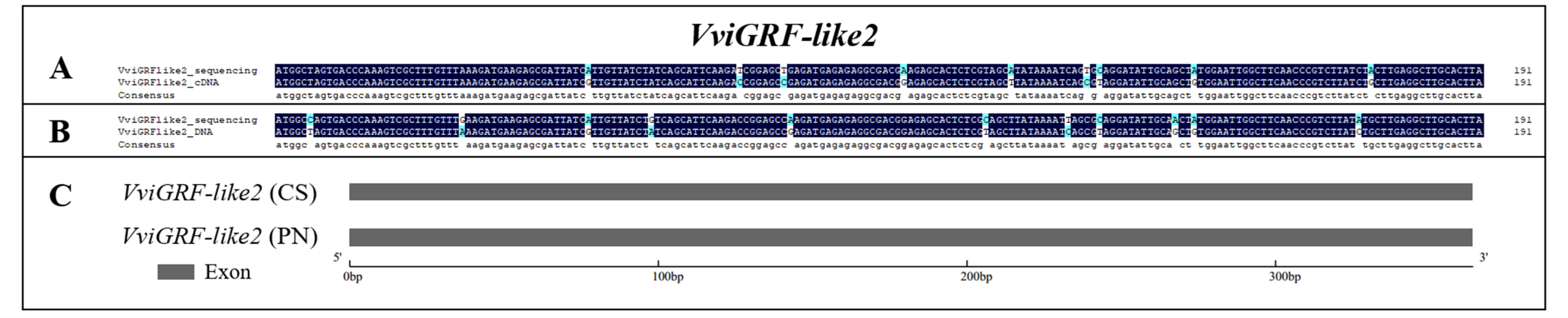
**

Supplement: Supplementary file 4 — Comparison of sequencing results and exon—intron structure of seven VviGRF genes. (A) cDNA Sequence alignment of VviGRFs. ‘_sequencing’ means cDNA of each VviGRF cloned from green stem in Cabernet Sauvignon. ‘_cDNA’ means reference sequences of each VviGRF cDNA in Point Noir. (B) DNA Sequence alignment of VviGRFs. ‘_sequencing’ means DNA of each VviGRF cloned from green stem in Cabernet Sauvignon. ‘_DNA’ means reference sequences of each VviGRF DNA in Point Noir. Sequence alignment analysis was performed by using DNAMAN version7. Sequences of seven VviGRFs in Cabernet Sauvignon were homologous with their reference sequence in Point Noir (Identity > 94%). (C) The exon—intron structure of seven VviGRFs. CS means sequencing gene structure in Cabernet Sauvignon, PN means reference gene structure in Point Noir. (DOC 18109 kb) [file 12864_2018_4955_MOESM4_ESM.doc]

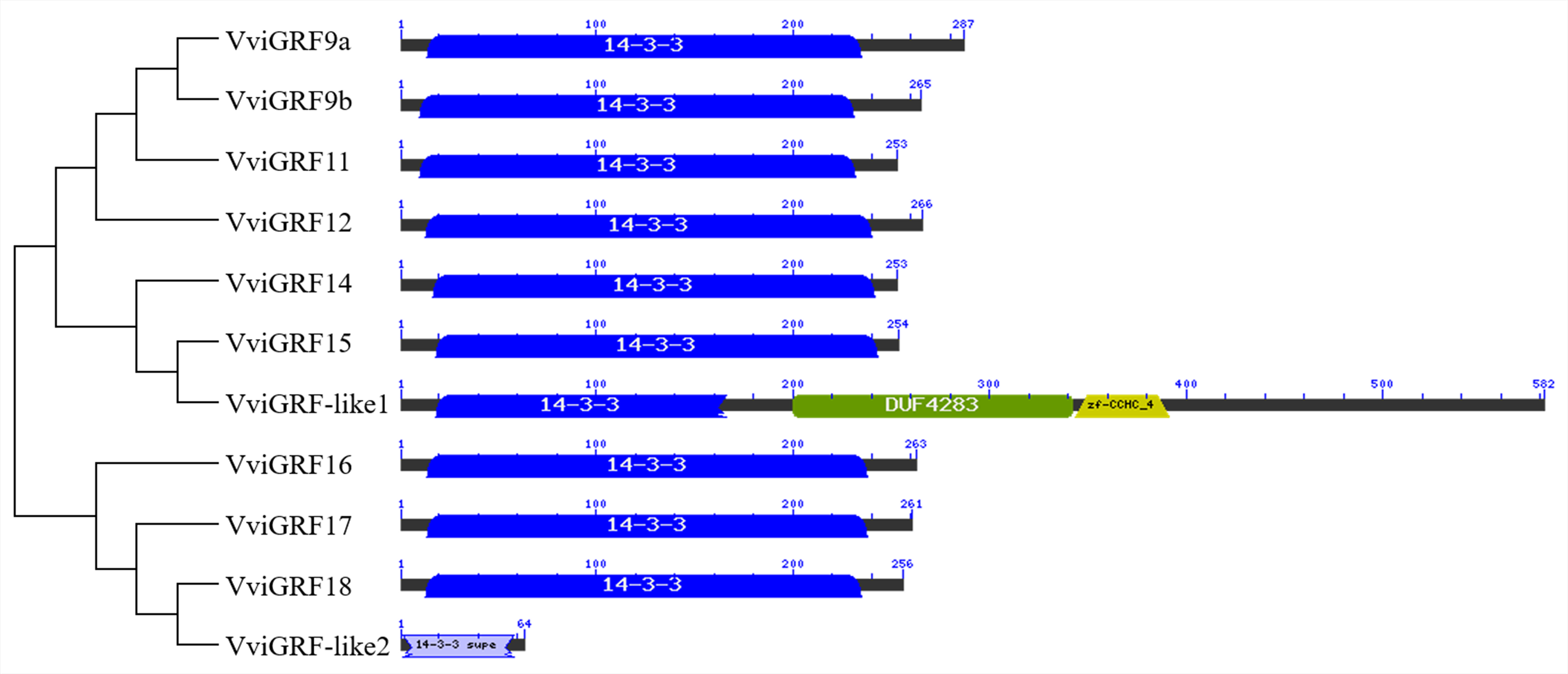

Supplement: Supplementary file 6 — The conserved domain analysis of VviGRF proteins. 14–3-3 domain was present in blue strips, and the jagged edges means incomplete N- or C-terminal. (TIF 679 kb) [file 12864_2018_4955_MOESM6_ESM.tif]

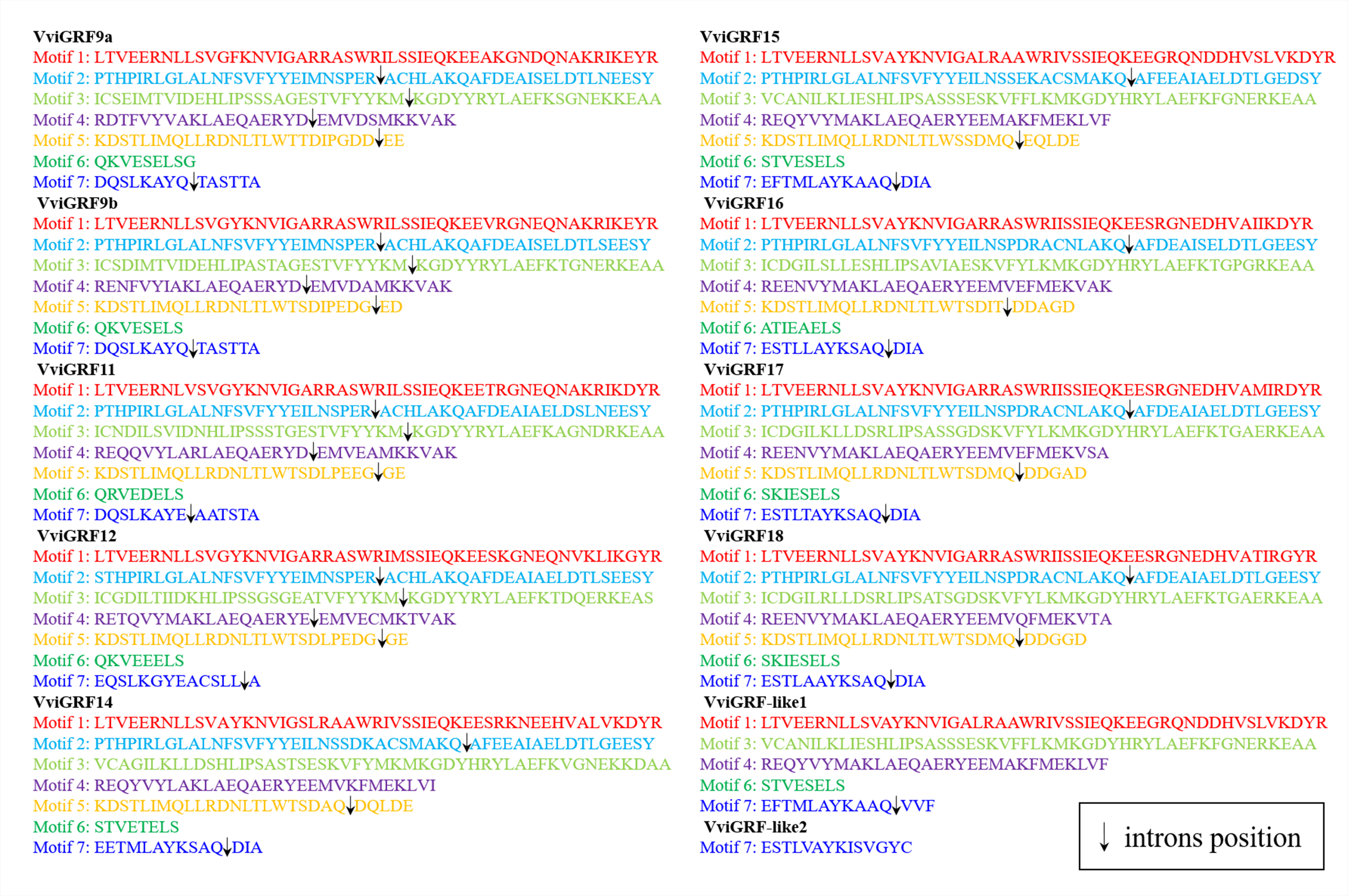

Supplement: Supplementary file 7 — Motif sequences of VviGRF proteins. Black arrow shows the introns position appeared in “exon-intron-exon” sequences. (TIF 5120 kb) [file 12864_2018_4955_MOESM7_ESM.tif]

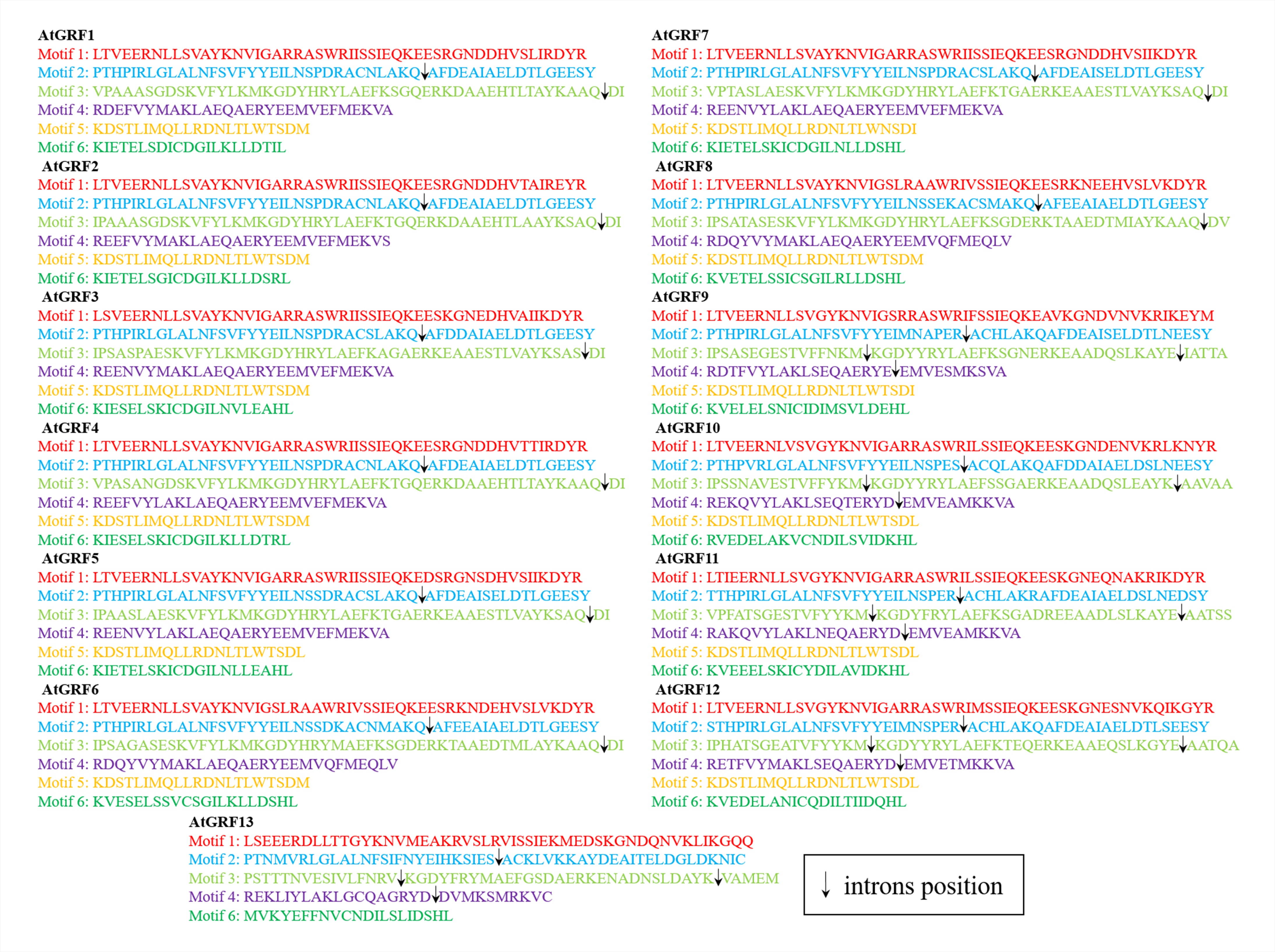

Supplement: Supplementary file 9 — Motif sequences of AtGRF proteins. Black arrow shows the introns position appeared in “exon-intron-exon” sequences. (TIF 6446 kb) [file 12864_2018_4955_MOESM9_ESM.tif]
