# Supplementary material for: Genome-wide identification and characterization of the 14–3-3 family in Vitis vinifera L. during berry development and cold- and heat-stress response
Source: BMC Genomics. 2018 Aug 2;19:579. doi: 10.1186/s12864-018-4955-8 (PMC6090852; doi:10.1186/s12864-018-4955-8)
Supplement: Supplementary file 2 — The organs presented in Fig. 6 from published data GSE36128. (DOC 63 kb) [file 12864_2018_4955_MOESM2_ESM.doc]

| **Additional file 2** |  |  |
| --- | --- | --- |
|  |  |  |
| **Sample/organ** | **Developmental stages collected** | **Abbreviations** |
| Bud | latent bud | Bud - L |
|  | winter bud | Bud - W |
|  | bud swell | Bud - S |
|  | bud burst (green tip) | Bud - B |
|  | bud after-burst (rosette of leaf tips visible) | Bud - AB |
| Inflorescence | young inflorescence (single flower in compact groups) | Inflorescence - Y |
|  | well developed inflorescence (single flower separated) | Inflorescence - WD |
| Flower | flowering begins (10% caps off) | Flower - FB |
|  | flowering (50% caps off) | Flower - F |
| Stamen | pool of stamens from undisclosed flowers at 10% and 50% open flowers | Stamen |
| Pollen | pollen from disclosed flowers at more than 50% open flowers | Pollen |
| Carpel | pool of carpels from undisclosed flowers at 10% and 50% open flowers | Carpel |
| Petal | pool of petals from undisclosed flowers at 10% and 50% open flowers | Petal |
| Tendril | young tendril (pool of tendrils from shoot of 7 leaves) | Tendril - Y |
|  | well developed tendril (pool of tendrils from shoot of 12 leaves) | Tendril - WD |
|  | mature tendril (pool of tendrils at fruit set) | Tendril - FS |
| Leaf | young leaf (pool of leaves from shoot of 5 leaves) | Leaf - Y |
|  | mature leaf (pool of leaves from shoot at fruit set) | Leaf - FS |
|  | senescencing leaf (pool of leaves at the beginning of leaf fall) | Leaf - S |
| Berry Pericarp | fruit set | Berry Pericarp - FS |
|  | post-fruit set | Berry Pericarp - PFS |
|  | véraison | Berry Pericarp - V |
|  | mid-ripening | Berry Pericarp - MR |
|  | ripening | Berry Pericarp - R |
|  | post-harvest withering I (1st month) | Berry Pericarp - PHWI |
|  | post-harvest withering II (2nd month) | Berry Pericarp - PHWII |
|  | post-harvest withering III (3rd month) | Berry Pericarp - PHWIII |
| Berry Skin | post-fruit set | Berry Skin - PFS |
|  | véraison | Berry Skin - V |
|  | mid-ripening | Berry Skin - MR |
|  | ripening | Berry Skin - R |
|  | post-harvest withering I (1st month) | Berry Skin - PHWI |
|  | post-harvest withering II (2nd month) | Berry Skin - PHWII |
| Berry Flesh | post-fruit set | Berry Flesh - PFS |
|  | véraison | Berry Flesh - V |
|  | mid-ripening | Berry Flesh - MR |
|  | ripening | Berry Flesh - R |
|  | post-harvest withering I (1st month) | Berry Flesh - PHWI |
|  | post-harvest withering II (2nd month) | Berry Flesh - PHWII |
| Seed | fruit set | Seed - FS |
|  | post-fruit set | Seed -PFS |
|  | véraison | Seed - V |
|  | mid-ripening | Seed – MR |
| Rachis | fruit set | Rachis-FS |
|  | post-fruit set | Rachis-PFS |
|  | véraison | Rachis-V |
|  | mid-ripening | Rachis-MR |
|  | ripening | Rachis-R |
| Stem | green stem | Stem - G |
|  | woody stem | Stem - W |
| Root | in *vitro* cultivation | Root |
| Seedling | pool of 3 developmental stages | Seedling |
| Total | 54 |  |
