# Supplementary material for: Genome-wide identification and characterization of the 14–3-3 family in Vitis vinifera L. during berry development and cold- and heat-stress response
Source: BMC Genomics. 2018 Aug 2;19:579. doi: 10.1186/s12864-018-4955-8 (PMC6090852; doi:10.1186/s12864-018-4955-8)
Supplement: Supplementary file 3 — The primer sequence for verifying exon—intron structure of seven VviGRF genes. (DOC 30 kb) [file 12864_2018_4955_MOESM3_ESM.doc]

**Additional file 3**

| V1 gene name | F-Primer | R-Primer |
| --- | --- | --- |
| *VviGRF9b* | AGAGAAAGTAGGGATCAGTAATGG | CTTCTAATTTCCGTCCGAGAG |
| *VviGRF11* | AGAGCAACAGGTTTACTTGG | CATTCCTCCATCCTTCTACGA |
| *VviGRF14* | GCAACTCGGACTTTACTGTG | GACATTCAAAGCCCACCTCC |
| *VviGRF15* | CAGAAACCCTAACCCGAGAG | CTGGTCCCAATGTTAAGGCAC |
| *VviGRF16* | ATGTTGTTGACCGAATCATCTC | GAACATTTCAACCCTGAGCAC |
| *VviGRF17* | CGAAGAGAACGTCTACATGG | ACAAGAATGCCTAGAAACTGAG |
| *VviGRF-like2* | ACAATCCTCCATCTGTGACGA | GCTCTTCATTCAGAGTGTCC |
