# Supplementary material for: Genome-wide identification and characterization of the 14–3-3 family in Vitis vinifera L. during berry development and cold- and heat-stress response
Source: BMC Genomics. 2018 Aug 2;19:579. doi: 10.1186/s12864-018-4955-8 (PMC6090852; doi:10.1186/s12864-018-4955-8)
Supplement: Supplementary file 5 — The primer sequence for qRT-PCR. (DOC 31 kb) [file 12864_2018_4955_MOESM5_ESM.doc]

**Additional file 5**

| V1 gene name | F-Primer | R-Primer |
| --- | --- | --- |
| *VviGRF9a* | ATGAACTCCCCTGAAAGGGC | GGTTTCCAGCTTCCAGCTCT |
| *VviGRF9b* | TGGAGACGATGAGGATGATTCC | AATTTCCGTCCGAGAGTGGC |
| *VviGRF11* | ACAGGTTTACTTGGCTAGGCT | TCCATGATGCCCTTCTTGCC |
| *VviGRF14* | AGGAGCATGTGGCTCTTGTC | GCCGAAGGTATCAGATGCGA |
| *VviGRF15* | ACATCCCATAAGGTTGGGGC | ACCCAATGTGTCCAGCTCAG |
| *VviGRF16* | GAAAGAGGCTGCAGAGAGCA | CCCCAGCCTTATTGGGTGAG |
| *VviGRF17* | AACTCACTGTCGAGGAACGG | GATCATGGCAACGTGGTCCTC |
| *VviGRF18* | CTACGTCCGGTGACTCGAAG | CTGTTGCAATATCCTGCGCC |
| *VviGRF-like2* | TTCGGCTATGGCTAGTGACC | GCTCCGGTCTTGAATGCTGA |
| *VviActin* | CTTGCATCCCTCAGCACCTT | TCCTGTGGACAATGGATGGA |
