# Supplementary material for: Genome-wide identification and characterization of the 14–3-3 family in Vitis vinifera L. during berry development and cold- and heat-stress response
Source: BMC Genomics. 2018 Aug 2;19:579. doi: 10.1186/s12864-018-4955-8 (PMC6090852; doi:10.1186/s12864-018-4955-8)
Supplement: Supplementary file 8 — Expression profile of 14–3-3 genes in V. vinifera cv. Muscat of Hamburg under cold stress from published data SRP018199. (DOC 28 kb) [file 12864_2018_4955_MOESM8_ESM.doc]

**Additional file 8**

| Gene ID | Accession Number | DEGs in *Vitis vinifera* cv. Muscat of Hamburg | | | | | | | | |
| --- | --- | --- | --- | --- | --- | --- | --- | --- | --- | --- |
|  |  | TPM in control |  | TPM in cold treatment |  | Log_Fold Change |  | *p*-value |  | FDR |
| GSVIVT01009141001 | XP_003634483 | 81.17783343 |  | 179.503 |  | 1.144851 |  | 9.97E-82 |  | 2.63E-77 |
| GSVIVT01012207001 | NP_001238407 | 12.75651668 |  | 33.64388 |  | 1.39911 |  | 1.22E-22 |  | 3.23E-18 |
