# Supplementary material for: Genome-wide identification and characterization of the 14–3-3 family in Vitis vinifera L. during berry development and cold- and heat-stress response
Source: BMC Genomics. 2018 Aug 2;19:579. doi: 10.1186/s12864-018-4955-8 (PMC6090852; doi:10.1186/s12864-018-4955-8)
Supplement: Supplementary file 10 — Detail information of grape 14–3-3 family. (DOC 39 kb) [file 12864_2018_4955_MOESM10_ESM.doc]

**Additional file 10**

| **Group** | **Gene** | **Locus ID** | **Chromosome Location** | **V0 protein** | **V0_Accession** |
| --- | --- | --- | --- | --- | --- |
|  |  |  |  |  |  |
| **Epsilon group** | *VviGRF9a* | VIT_10s0003g01240 | chr10: 2568369-2571848 | GSVIVT01021210001 | CBI19165 |
|  | *VviGRF9b* | VIT_19s0014g01420 | chr19: 1489168-1494033 | GSVIVT01014210001 | CBI30682 |
|  | *VviGRF11* | VIT_18s0001g05720 | chr18: 4453327-4457820 | GSVIVT01009037001 | CBI20197 |
|  | *VviGRF12* | VIT_01s0011g00620 | chr1: 563253-566683 | GSVIVT01012207001 | CBI18706 |
| **Non-epsilon group** | *VviGRF14* | VIT_18s0001g07240 | chr18: 5477686-5481158 | GSVIVT01009141001 | CBI19238 |
|  | *VviGRF15* | VIT_00s0199g00190 | chrUn: 11210066-11213944 | GSVIVT01003608001 | CBI18710 |
|  | *VviGRF16* | VIT_14s0006g03230 | chr14: 21820857-21823454 | GSVIVT01031070001 | CBI19195 |
|  | *VviGRF17* | VIT_18s0001g06330 | chr18: 4742712-4744730 | GSVIVT01009076001 | CBI33672 |
|  | *VviGRF18* | VIT_07s0191g00090 | chr7: 14850330-14852315 | GSVIVT01003421001 | CBI33141 |
|  | *VviGRF-like1* | VIT_00s0199g00140 | chrUn: 11185021-11188247 | GSVIVT01003603001 | CBI27277 |
|  | *VviGRF-like2* | VIT_02s0033g00780 | chr2: 15461190-15461553 | — | — |
